# Whole exome sequencing reveals inherited and *de novo* variants in autism spectrum disorder: a trio study from Saudi families

**DOI:** 10.1038/s41598-017-06033-1

**Published:** 2017-07-18

**Authors:** Bashayer Al-Mubarak, Mohamed Abouelhoda, Aisha Omar, Hesham AlDhalaan, Mohammed Aldosari, Michael Nester, Hussain. A. Alshamrani, Mohamed El-Kalioby, Ewa Goljan, Renad Albar, Shazia Subhani, Asma Tahir, Sultana Asfahani, Alaa Eskandrani, Ahmed Almusaiab, Amna Magrashi, Jameela Shinwari, Dorota Monies, Nada Al Tassan

**Affiliations:** 1 Behavioral Genetics unit, Department of Genetics, King Faisal Specialist Hospital & Research Center, P.O. Box 3354, Riyadh, 11211 Saudi Arabia; 2 Saudi Human Genome Program, King Abdulaziz City for Science and Technology, Riyadh, Saudi Arabia; 3 Department of Genetics, King Faisal Specialist Hospital & Research Centre, P.O. Box 3354, Riyadh, 11211 Saudi Arabia; 4 Center for Autism Research, King Faisal Specialist Hospital & Research Centre, P.O. Box 3354, Riyadh, 11211 Saudi Arabia; 5 Department of Neurosciences, King Faisal Specialist Hospital & Research Centre, P.O. Box 3354, Riyadh, 11211 Saudi Arabia; 6 Pediatric Department, King Faisal Specialist Hospital & Research Centre, P.O. Box 3354, Riyadh, 11211 Saudi Arabia

## Abstract

Autism spectrum disorder (ASD) is a complex neurodevelopmental disorder with genetic and clinical heterogeneity. The interplay of *de novo* and inherited rare variants has been suspected in the development of ASD. Here, we applied whole exome sequencing (WES) on 19 trios from singleton Saudi families with ASD. We developed an analysis pipeline that allows capturing both *de novo* and inherited rare variants predicted to be deleterious. A total of 47 unique rare variants were detected in 17 trios including 38 which are newly discovered. The majority were either autosomal recessive or X-linked. Our pipeline uncovered variants in 15 ASD-candidate genes, including 5 (*GLT8D1*, *HTATSF1*, *OR6C65*, *ITIH6* and *DDX26B*) that have not been reported in any human condition. The remaining variants occurred in genes formerly associated with ASD or other neurological disorders. Examples include *SUMF1*, *KDM5B* and *MXRA5* (Known-ASD genes), *PRODH2* and *KCTD21* (implicated in schizophrenia), as well as *USP9X* and *SMS* (implicated in intellectual disability). Consistent with expectation and previous studies, most of the genes implicated herein are enriched for biological processes pertaining to neuronal function. Our findings underscore the private and heterogeneous nature of the genetic architecture of ASD even in a population with high consanguinity rates.

## Introduction

Delineating the genetic architecture of Autism Spectrum Disorder (ASD) is like finding the way in a labyrinth. Despite the rapid advancements in genetic methods, our ability to identify a common pathway for neuropsychiatric disorders with a therapeutic or diagnostic potential is lagging behind^1^. This is best exemplified by ASD, for which delivering a near complete genetic picture is hindered by many factors. Examples of such factors include: (i) extensive clinical and genetic heterogeneity (in excess of 600 genes have been implicated in ASD, thus far)^2^; (ii) the absence of generalizable genetic risk factors as most of the mutations are extremely rare or private in nature; (iii) the variability in diagnosis and data analysis methodology; and finally (iv) the precise function of many of ASD-candidate genes and therefore their possible impact on the central nervous system remains largely undetermined^3, 4^. All these factors present a substantial challenge, yet incremental progress has and continues to be made towards identifying ASD-risk loci.

Over the past 15 years, efforts were centered on interrogating the role of structural alterations through the study of chromosomal abnormalities and copy number variations (CNVs) in ASD individuals. Key advances have emerged from such studies, for instance, the identification of cytogenetic abnormalities at multiple loci with chromosomes 15q11–13 and 16p11.2 being the most frequent^5^, as well as recurrent CNVs involving genes like, *NRXN1*, *SHANK3* and *PARK2* ^6^. However, a causal relationship is often hard to prove for a number of reasons: (i) such events normally span large regions making it difficult to discern the role of single genes in the disorder; (ii) apparently identical alterations have variable phenotypic outcomes or expressivity; (iii) some of these changes are observed in typically developing individuals; (iv) very low rate of replication, as recurrent individual CNVs were observed only in less than 1% of the cases^7^.

In the more recent years and with the advent of next-generation sequencing (NGS) technologies, the focus shifted towards investigating the role of inherited and *de novo* point mutations. This move was triggered by the observation that potentially risk-conferring structural alterations occurred only in (1–20%) of the cases^7^ and that they probably require other genetic or non-genetics factors for the development of the disease. The increasing availability of whole-genome and –exome sequencing platforms to researchers have accelerated the identification of additional risk factors in previously recognized as well as unrecognized ASD-associated genes^8–18^. However, most of these studies have focused on the role of either *de novo* or inherited variants^3, 8, 9, 11–16, 19, 20^ and much fewer were designed to interrogate both types^10, 18, 21, 22^. As there is a growing appreciation for the important part transmitted and *de novo* mutations play in ASD, more studies assessing both types of events within a given cohort are needed to understand their precise role.

**Figure 1:**
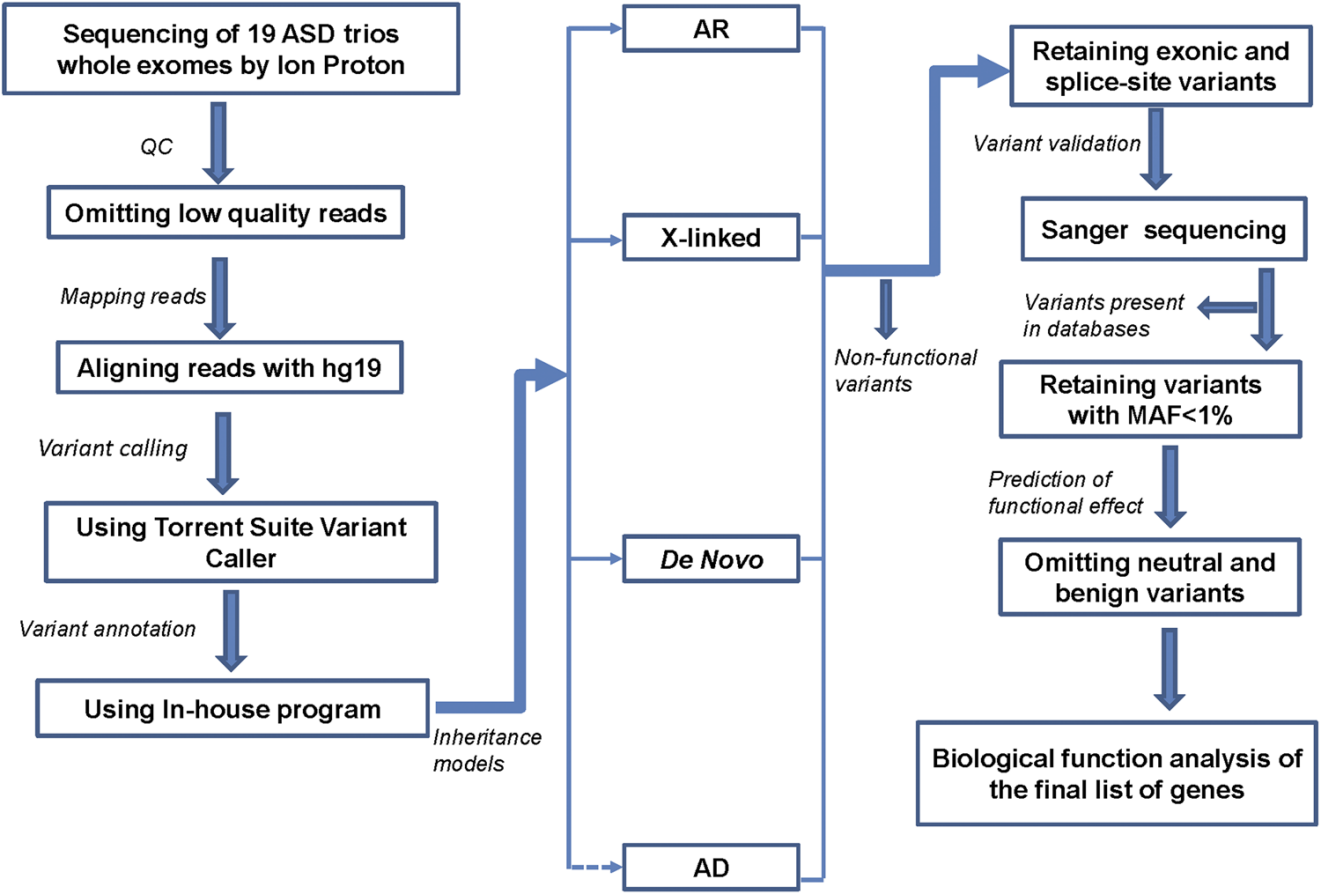
Schematic illustrating the steps of WES data analysis pipeline employed in this study. The multi-step analysis procedure involves eliminating low quality reads, variant mapping to human reference genome (hg19), variant calling and annotation, grouping variants according to inheritance models followed by variant prioritization and validation, and finally biological functions analysis was carried out on the final list of genes. AR, autosomal recessive; AD, autosomal dominant; QC, quality control. Dashed arrow indicates that the model was applied when all three main models failed to discover candidate variant(s).

In this study, we implemented whole exome sequencing (WES) in an attempt to identify risk genes/rare variants in a cohort of 19 case-parent trios from singleton families with ASD from Saudi Arabia. Enrichment for inherited causes are predicted in highly inbred populations^17^ as are sporadic (*de novo*) mutations in families with apparently “unaffected” parents^8–10, 12, 13, 18, 20, 23, 24^. We therefore, devised an analysis pipeline that enabled us to detect *de novo* as well as inherited variants in autosomal or X-linked genes (Fig. 1). Using this approach, rare variants were ascertained in 17 of the 19 trios (~90%).We found that most of the probands carried at least 2 unique rare variants occurring in 47 different genes. To the best of our knowledge 15 of the identified genes were not implicated before in ASD and the remaining were previously reported in ASD or other neuropsychiatric/neurodevelopmental disorders. Examples include *PRODH2* and *KCTD21* (implicated in schizophrenia), *USP9X* and *SMS* (implicated in intellectual disability), *TRIM9* and *KDM5B* (known-ASD genes), and others such as *AGL* and *MOGS* (candidate genes involved in energy metabolism and immune responses, respectively).

## Results

### Sequencing and Alignment Quality

Supplementary Table S1 summarizes the quality of the sequencing and the read mapping steps. It shows that the sequencing achieved good coverage of the target regions (98% average total coverage at 1X and 95% average total coverage at 20X) with enough depth (223X; i.e. each base in the target region is covered by 223 reads on average). We detected an average of 29120.333 total variants comprised of 27466.807 Single Nucleotide Variants (SNVs) and 1653.526 indels per exome (Supplementary Table S2). After comparing the trios, a list of candidate variants was sent to Sanger sequencing for confirmation. The frequency of the confirmed variants in international databases (ExAC, 1000 Genomes Project) and the local the Saudi Human Genome Program Database (SHGP; 2379 local ethnically matching exomes) was also considered. We counted 29 false positives of which 20 were SNVs and 9 were indels as a result of low quality calls.

Reported familial relations history was assessed with three different relatedness tests (described in the methods). The results confirm correct parenthood for all our trios (Supplementary Table S3) as higher scores for parent-child pair were observed by all three methods. For our dataset, the results using the *A*_*jk*_ statistics and shared homozygosity were more confirmative than the ones generated by KING program.

### Detected SNVs and Indels

The analysis framework (described in methods) developed in this study allows for comprehensive investigation of the genetic architecture of ASD (Fig. 1). It is designed to capture not only *de novo* variants, but also those with autosomal or X-linked mode of transmission in unknown or previously described ASD-linked genes. This analysis approach is more suited for complex disorders with high genetic heterogeneity and it may offer much needed explanation for the increased ASD heritability^15^.

**Figure 2:**
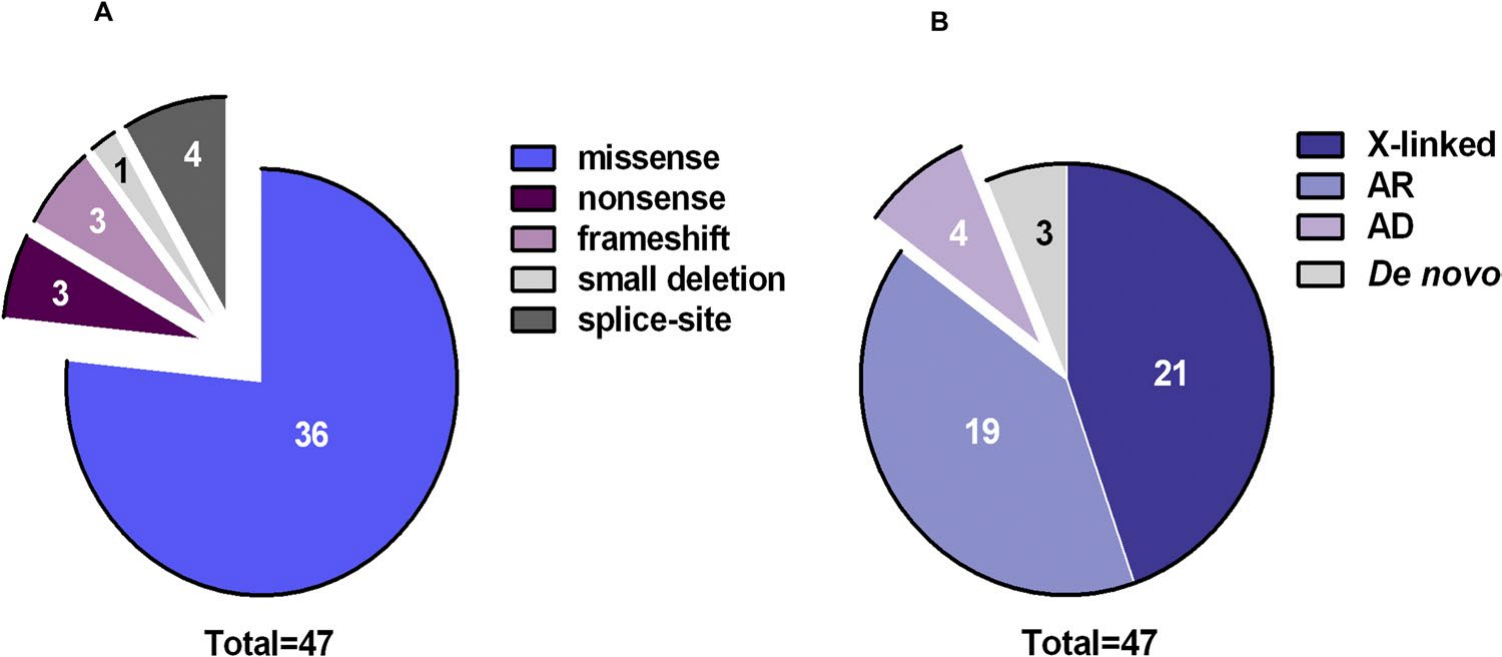
Effect and mode of transmission of the validated variants. Pie charts illustrating the distribution of the confirmed variants found in all probands according to their effect (**A**) or mode of transmission (**B**). AR: autosomal recessive. AD: autosomal dominant.

Using the 4 main inheritance models (refer to methods) a number of variants was detected in each sample except two (ASD-52 and ASD-55). From the variants that have passed all filtering steps, 44 were considered to be deleterious by at least one prediction tool and were confirmed as being true positives for presence of the variant by Sanger sequencing. The majority of the validated variants were missense, while the remaining were either frameshifts, splice-site changes or nonsense and only one small deletion was detected (Fig. 2A). As for the mode of transmission, 3 were *de novo*, 4 were autosomal dominant, 21 were found in X-linked genes, and the remaining 19 were inherited in an autosomal recessive manner (Fig. 2B). Only 9 of the verified variants were previously reported (in dbSNP with MAF < 1%) (*MOGS*, rs370842409; *ITIH2*, rs748626881; *OR6C6*, rs748626881; *GPKOW*, rs782015404, *TRIM9*, rs748897524; *NEB* rs375412223; *BOC*, rs752313669; *SSTR3*,rs577113986; *PRODH2*,rs370842409), while the remaining 38 variants were novel. Altogether, we have identified 47 rare variants in 47 different genes including 15 ASD-candidate genes (Tables 1 and 2).

**Table 1:** Summary of the genes with confirmed rare variants detected in this study.

Gene	OMIM/HGNC	Gene function	Biological process(s)	PANTHER Protein Class	Human disease	Reported link to ASD	Autism databases		Reported link to other neurological disorders
							SFARI Gene	Autism KB	
*ACE2*	300335/13557	Angiotensin converting enzyme 2	Cell signaling cascade (Renin angiotensin system)	NA	NI	Yes	No	No	No
*AGL*	610860/321	Glycogen debranching enzyme necessary for glycogenolysis	Mitochondrial function/Energy metabolism	Glycosyltransferase/glycosidase	Glycogen storage disease IIIa and b	No	No	No	No
*APC2*	612034/24036	Tumor suppressor involved in microtubules stabilization	Neuronal function/Development	NA	^a^ Sotos syndrome-like features	Yes	No	Yes	Patients with Sotos syndrome-like features also had intellectual disability
*ARSH*	300586/32488	Arylsulfatase H	Unknown	NA	NI	Yes	No	Yes	No
*ASB9*	300890/17184	E3 ubiquitin-ligase involved in regulating the levels of creatine kinase B	Protein quality control	NA	NI	Yes	No	Yes	No
*ATP2B3*	300014/816	The plasma membrane Ca^2+^ ATPase acts as a route for cellular calcium extrusion	Cell signaling cascade (Calcium homeostasis)	Cation transporter/ion channel/hydrolase	X-linked congenital cerebellar ataxia	Yes	No	Yes*	Possible association with ID
*AVPR2*	300538/897	Arginine-vassopressin receptor 2	Water homeostasis	G-protein coupled receptor	Nephrogenic diabetes insipidus	Yes	No	Yes	In dizygotic twins with nephrogenic diabetes insipidus and intellectual disability (ID)
*BOC*	608708/17173	An atypical receptor for Sonic Hedgehog regulating synapse formation	Neuronal function/Development	Immunoglobulin superfamily cell adhesion molecule	NI	Candidate gene in a patient with 3q13.31 microdeletion presenting with neurodevelopmental phenotype and autism	No	Yes	No
*CELSR2*	604265/3231	Involved in axonal guidance	Neuronal function/Development	G-protein coupled receptor/cadherin	NI	No	No	No	SZ
*CEP152*	613529/29298	Maintenance of genomic integrity and centrosome duplication	Cell division or differentiation	Signaling molecule	Seckel syndrome and primary microcephaly	Yes	No	Yes	No
*CRY1*	601933/2384	Circadian clock gene	Sleep regulation	DNA photolyase/lyase	NI	Sleep problems are common in autistic children	No	Yes	Possible association with unipolar depression
*CXORF30/CFAP47*	NA/6708	Unknown	Unknown	NA	NI	Yes	No	Yes	No
*DDX26B/INTS6L*	NA/27334	Unknown	Unknown	NA	NI	No	No	No	No
*DNAJC13*	614334/30343	Component of the endosomal recycling system	Protein quality control	NA	Parkinson’s disease	No	No	No	Parkinson’s disease
*DUSP3*	600183/3069	Dual-specificity phosphatase	Cell signaling cascade (MAPK signaling)	NA	NI	Yes	No	Yes*	No
*FGF5*	165190/3683	Growth factor with neurotrophic activity	Neuronal function/Development	Growth factor	NI	Yes	No	Yes	No
*FLNA*	300017/3754	Actin-binding protein that may contribute to dentrites development	Neuronal function/Development	Non-motor actin binding protein	NI	Yes	No	Yes	No
*GLT8D1*	NA/24870	Unknown	Unknown	NA	NI	No	No	No	No
*GPKOW*	NA/30677	Component of the human spliceosome	Gene regulation (mRNA splicing)	NA	NI	Yes	No	Yes	No
*HSPBP1*	612939/24989	Hsp70-binding protein involved in protection against stress-induced damage	Protein quality control	NA	NI	Autism with FX mutation and chr15 dup	No	Yes	Autism with FX mutation and chr15 dup
*HTATSF1*	300346/5276	Transcription-splicing factor	Gene regulation (mRNA splicing)	NA	NI	No	No	No	No
*IDS*	300823/5389	Lysosomal degradation of glycosami-noglycans	Protein quality control	Hydrolase	Mucopolysa-ccharidosis type II	Yes	No	Yes	No
*ITIH2*	146640/6167	Plasma protease inhibitor involved in the stabilization of the extracellular matrix	Unknown	NA	NI	Yes	No	Yes	No
*ITIH6*	NA/28907	Unknown	Unknown	NA	NI	No	No	No	No
*KCTD21*	NA/27452	Cullin-3 adaptor protein required for HDAC1 ubiquitination and degradation	Gene regulation/Protein quality control	Enzyme modulator	Medulloblastoma	Yes	No	Yes	SZ
*KDM5B*	605393/18039	A demethylase involved in genome stability maintenance, and cell fate determination	Gene regulation/Cell differentiation	Zinc finger transcription factor	NI	*De novo* mutations identified in ASD patients	Yes*	Yes	Non-syndromic ID
*MAN1B1*	604346/6823	ER-located endoplasmic enzyme involved in targeting misfolded glycoproteins for ER-associated degradation	Protein quality control	Chaperone	Congenital disorders of glycosylation	Yes	No	Yes	ID
*MAOB*	309860/6834	Deamination of a number of neurotransmitters	Neuronal function/Development	DNA methyltransferase; oxidase; DNA binding Protein	NI	No	Yes	Yes^#^	SZ
*MOGS*	601336/24862	Normal glycans processing	Immune responses	NA	Congenital disorders of glycosylation type II B	No	No	No	No
*MXRA5*	300938/7539	Proteoglycan involved in extracellular matrix remodeling and cell-cell adhesion	Neuronal function/Development	Immunoglobulin superfamily cell adhesion molecule	NI	Mutations identified in three unrelated multiplex ASD families	No	Yes	No
*NEB*	161650/7720	Actin regulator	Neuronal function/Development	NA	Nemaline myopathy 2	No	No	No	Mutations in this gene were identified in patients exhibiting ID and epilepsy with other symptoms
*NGF*	162030/7808	Trophic factor that has a role in behavior and brain development	Neuronal function/Development	Neurotrophic factor	Hereditary sensory and autonomic neuropathy type V	Yes	No	Yes	No
*NLRP2*	609364/22948	Part of the inflammasome complex in astrocytes and acts as an inhibitor of NF-κB	Immune responses	Transcription co-factor/Nucleic acid binding	Beckwith-Wiedemann syndrome	Yes	No	Yes	No
*NT5DC1*	NA/21556	Unknown	Unknown	NA	NI	No	No	No	Bipolar disorder
*OR6C65*	NA/31295	Olfactory receptor	Olfaction	NA	NI	Impaired olfaction was reported in ASD	No	Yes	No
*PDK3*	300906/8811	Negative regulator of pyruvate dehydrogenase	Mitochondrial function/Energy metabolism	Protein kinase	X-linked dominant Charcot-Marie-Tooth disease	Yes	No	Yes	No
*PLP1*	300401/9086	Major constituent of the CNS myelin	Neuronal function/Development	Myelin protein	Pelizaeus-Merzbacher disease and spastic paraplegia type 2	No	No	No	Microdeletion of Xq22 encompassing *PLP1* in female patients with ID and behavioral problems
*PRODH2*	616377/17325	Catalyzes proline degradation	Neuronal function/Development	Oxidase	NI	No	No	No	SZ
*RPS6KA6*	300303/10435	Ribosomal protein S6 kinase A6	Cell signaling cascade (MAPK signaling)	Non-receptor serine/threonine protein Kinase;transfer/carrier protein/annexin/calmodulin	NI	Yes	No	Yes*	Possible role in nonspecific X-linked ID
*SEMG2*	182141/10743	Human seminal plasma protein	Semen function	NA	NI	Yes	No	Yes	No
*SMS*	300105/11123	Synthesis of spermine that has a modulatory effect on NMDAR	Neuronal function/Development	Transferase	X-linked intellectual disability (Snyder-Robinson type)	Yes	No	Yes	X-linked intellectual disability (Snyder-Robinson type)
*SSTR3*	182453/11332	G protein-coupled receptor somatostatin receptor 3 with a role in synapse formation	Neuronal function/Development	G-protein coupled receptor	NI	Yes	No	Yes	No
*SSX3*	300325/11337	Unknown	Unknown	NA	NI	Yes	No	Yes	No
*SUMF1*	607939/20376	Activator of steroid sulfatases	Neuronal function/Development	NA	NI	Structural variants detected in ASD	No	Yes	SZ and multiple sclerosis
*TRIM9*	606555/16288	E3 ubiquitin-ligase that plays a role in axonal branching	Neuronal function/Development	Ubiquitin-protein ligase	NI	Identified as a putative causative gene in one ASD case	No	No	Atypical psychosis
*USP9X*	300072/12632	Deubiquitinating enzyme	Neuronal function/Development	NA	X-linked intellectual disability	Yes	No	Yes^#^	X-linked intellectual disability
*ZNF630*	300819/28855	Unknown	Unknown	NA	NI	Yes	No	Yes	No

*Nonsynonymous variant(s) were reported. ^#^Synonymous variant(s) were reported. Schizophrenia: SZ. Intellectual disability: ID. Fragile-X: FX. NI: Not identified. NA: Not available. For more details refer to supplementary information.

**Table 2:** Rare variants identified in each trio.

Proband ID (Gender)	Mode of Inheritance	Identified Variant			Genotype				CADD (scaled)	ExAC	MAF		ROH
		Gene	Base Change	Amino Acid Change	Proband	Other family members				pLI/Z score	SHGP LC database	ExAC/1000 Genomes project	
						UF	UM	US/(Gender)					
ASD-9 (M)	AD	*AGL*	^#^c.294-2A > T	NA	A/T	A/T	A/A	NA	24.9	0.00/−2.23	0.0037	0/0	NA
	AD	*APC2*	^#^c.1207 + 3G > C	NA	G/C	G/C	G/G	NA	12.21	1.00/4.81	0	0.0002/0	NA
ASD-16 (F)	AR	*MAN1B1*	c.1897G > T	p.V633F	T/T	G/T	G/T	NA	26.5	0.00/−1.01	0	0.00001656/0	chr9:139943549–141071552
	AR	*KCTD21*	c.152G > A	p.R51H	A/A	G/A	G/A	NA	34	0.29/0.80	0	0.0001/0	chr11:74862391–77920563
	AR	*DUSP3*	c.439G > A	p.V147I	A/A	G/A	G/A	NA	24	0.74/1.28	0	0.00002476/0	chr17:41332822–42248346
	X-linked	*CXorf30/CFAP47*	c.8943G > A	p.M2981I	G/A	Hemi (A)	G/G	NA	13.16	No score	0	0/0	NA
	X-linked	*SSX3*	c.268C > A	p.R90S	C/A	Hemi (A)	C/C	NA	0.256	0.15/−1.08	0.0008	0/0	NA
	X-linked	*AVPR2*	c.739–750 delCGCCGCAGGGGA	p.247-250del	Het	Homo	WT	NA	NA	0.12/1.61	0.0009	0.0021/0.000529801	NA
ASD-17 (M)	AR	*CRY1*	c.272G > A	p.W91X	A/A	G/A	G/A	G/A (M)	40	0.00/1.46	0	0/0	chr12:99640557–107713511
	AR	*NLRP2*	c.523T > C	p.W175R	C/C	T/C	T/C	T/T (M)	0.119	0.00/−1.98	0.0033	0.000008239/0	NA
	X-linked	*ACE2*	c.1235C > A	p.A412E	Hemi (A)	Hemi (C)	C/A	Hemi (A) (M)	25.3	1.00/0.98	0.0038	0/0	NA
ASD-18 (M)	AR	*GLT8D1*	^#^c.811_812 + 2delGAGT	p.E271K	Homo	Het	Het	NA	35	0.00/0.54	0.00168	0/0	chr3:52720080–53269046
	AR	*DNAJC13*	c.5594A > G	p.N1865S	G/G	A/G	A/G	NA	18.38	1.00/1.04	0.00042	0/0	chr3:129800895–133486958
	AR	*OR6C65*	c.766delA	p.M256fs	Homo	Het	Het	NA	27.6	0.00/−1.37	0.00042	0.00001648/0	chr12:53647373–56089357
	AR	*HSPBP1*	c.1016A > C	p.E339A	C/C	A/C	A/C	NA	26.7	0.01/0.79	0.00042	0/0	chr19:55525894–56047448
	X-linked	*ATP2B3*	c.1678C > G	p.P560A	Hemi (G)	Hemi (C)	C/G	NA	3.822	1.00/3.24	0	0.0000129/0.00001144	NA
ASD-19 (M)	X-linked	*MXRA5*	c.28C > T	p.L10F	Hemi (T)	Hemi (C)	C/T	NA	24.1	0.38/−1.92	0.0012	0/0	NA
	X-linked	*ITIH6*	c.1153A > G	p.S385G	Hemi (G)	Hemi (A)	A/G	NA	0.411	0.00/−3.80	0.0021	0/0	NA
	*De novo*	*KDM5B*	c.2265C > A	p.Y755X	C/A	C/C	C/C	NA	35	0.00/1.99	0	0.00003295/0	NA
ASD-21 (M)	AR	*NT5DC1*	c.1114T > C	p.S372P	C/C	T/C	T/C	NA	28.1	0.33/−0.27	0.0012	0/0	chr6:116442525–116574577
	AR	*TRIM9*	c.1643G > A	p.R548H	A/A	G/A	G/A	NA	15.5	0.99/4.12	0	0.0000106/0	chr14:51225132–51716467
ASD-24 (M)	X-linked	*GPKOW*	c.482C > T	p.A161V	Hemi (T)	Hemi (C)	C/T	Hemi (T) (M)	9.839	0.98/0.42	0.0037	0.00001368/0	NA
	X-linked	*HTATSF1*	c.894T > G	p.F298L	Hemi (G)	Hemi (T)	T/G	Hemi (G) (M)	24	0.97/1.80	0	0/0	NA
	*De novo*	*MOGS*	c.832_833delAA	p.K278Efs*5	Het	WT	WT	WT (M)	27	0.00/0.66	0	0.000008359/0	NA
ASD-37(M)	X-linked	*IDS*	c.525T > A	p.D175E	A/A	Hemi (T)	T/A	T/A (F)	12.51	0.98/1.18	0.0012	0/0	NA
ASD-38 (M)	X-linked	*ASB9*	c.310A > C	p.T104P	Hemi (C)	Hemi (A)	A/C	NA	25.6	0.89/0.44	0.0008	0/0	NA
	X-linked	*PDK3*	^#^c.248 + 3A > G	_	Hemi (G)	Hemi (A)	A/G	NA	15.91	0.88/2.64	0.0021	0/0	NA
	X-linked	*PLP1*	c.328G > A	p.G110S	Hemi (A)	Hemi (G)	G/A	NA	28.1	0.90/2.45	0	0/0	NA
ASD-39 (M)	AD	*NEB*	c.5939T > C	p.L1980S	T/C	T/T	T/C	NA	25	0.00/−4.04	0.00084	0.0003/0	NA
ASD-40 (M)	AR	*BOC*	c.1624G > A	p.G542R	A/A	G/A	G/A	NA	28.6	0.00/0.77	0	0.000008237/0	chr3:112546306–113377505
	AR	*SSTR3*	c.724G > C	p.A242P	C/C	G/C	G/C	NA	25.6	0.04/1.45	0	0.0003/0.000199681	chr22:32756744–37603744
ASD-43 (M)	AR	*NGF*	c.361C > T	p.R121W	T/T	C/T	C/T	NA	28.3	0.70/0.92	0.00084	0/0	chr1:114301335–116927468
	AR	*SUMF1*	c.710A > G	p.Q237R	G/G	A/G	A/G	NA	26.3	0.04/−0.62	0	0/0	chr3:439963–4856234
ASD-52 (M)	No candidate genes												
ASD-55 (M)	No candidate genes												
ASD-58 (M)	X-linked	*MAOB*	c.392G > T	p.S131I	Hemi (T)	Hemi (G)	G/T	NA	22.4	0.97/1.38	0.00042	0/0	NA
	X-linked	*ZNF630*	c.77A > T	p.N26I	Hemi (T)	Hemi (A)	A/T	NA	15.35	0.00/−1.42	0	0/0	NA
	*De novo*	*PRODH2*	c.625G > C	p.G209R	G/C	G/G	G/G	NA	0.461	0.00/−0.22	0	0.000008276/0	NA
ASD-64 (F)	X-linked	*USP9X*	c.3803A > G	p.Y1268C	A/G	Hemi (A)	A/G	NA	25.2	1.00/6.35	0.00042	0/0	NA
	X-linked	*RPS6KA6*	c.1535A > G	p.Q512R	A/G	Hemi (G)	A/A	NA	17.14	0.74/1.70	0.0025	0/0	NA
	X-linked	*DDX26B*/*INTS6L*	c.1304A > T	p.E435V	A/T	Hemi (T)	A/A	NA	27.8	1.00/1.74	0.00084	0/0	NA
ASD-66 (M)	AD	*SEMG2*	c.500G > A	p.W167X	G/A	G/G	G/A	NA	20.4	0.00/−2.36	0.0058	0.000008241/0	NA
ASD-69 (M)	AR	*CELSR2*	c.8185G > C	p.D2729H	C/C	G/C	G/C	G/C (M)	25.8	1.00/3.61	0	0/0	chr1:109395105–109816176
	AR	*ITIH2*	c.1863_1864insTATT	p.V622Yfs*17	Homo	Het	Het	WT (M)	24.3	0.00/−0.95	0	0.00007417/0	chr10:3200292–13337588
	AR	*CEP152*	c.1718A > C	p.D573A	C/C	A/C	A/C	A/C (M)	11.88	1.00/−1.59	0	0/0	chr15:45392075–49284771
	X-linked	*ARSH*	c.1124T > C	p.I375T	Hemi (C)	Hemi (T)	T/C	Hemi (C) (M)	22.3	0.00/−0.13	0	0/0	NA
ASD-73 (M)	AR	*FGF5*	c.251C > T	p.S84L	T/T	C/T	C/T	NA	24.3	0.00/−0.58	0	0/0	chr4:80977078–88535895
	X-linked	*SMS*	c.424C > T	p.L142F	Hemi (T)	Hemi (C)	C/T	NA	28.9	0.92/2.30	0	0/0	NA
	X-linked	*FLNA*	c.7079T > C	p.V2360A	Hemi (C)	Hemi (T)	T/C	NA	23.2	1.00/4.95	0.00042	0/0	NA

^#^PredictSNP2 score for splice site variants is “Deleterious” with expected accuracy of 90% or above. UF: unaffected father. UM: unaffected mother. US: unaffected sibling. ROH: region of homozygosity. NA: not applicable. CADD v1.3.

### *De novo* variants

*De novo* germline mutations arising spontaneously during meiosis have long been known to confer risk of ASD. This knowledge was inferred largely from studies assessing *de novo* CNVs in ASD cases from simplex or multiplex families^25–31^, however, structural variants seem to account for a small fraction of the cases^28, 32–34^, shifting the focus to *de novo* point mutations and their contribution to risk, which is a line of enquiry we and others have perused^8, 9, 11, 13, 20, 24^.

Among the 17 probands, 3 (17.6%) were found to carry rare *de novo* SNVs. These confirmed *de novo* events were identified in different genes including one with previous association with ASD: two of these events were predicted to result in a premature truncation, and the remaining was a missense rare variant (p.G209R) affecting *PRODH2*, found in proband ASD-58. The two nonsense variants were: (i) (p.Y755X) affecting *KDM5B* identified in ASD-19 and (ii) a frameshift insertion of two adenine bases creating a premature stop codon (p.K278Efs*5) in *MOGS* that was detected in proband ASD-24 (Table 2).

### Autosomal variants

Despite the excess and well-recognized importance of *de novo* mutations, only a fraction of ASD cases could be accounted for by these mutational events^8, 17^, while cases bearing inherited events couldn’t be fully explained by this class of genetic alteration. The high heritability of ASD^15^ underscores the need to explore transmitted variants that are often overlooked in many of the existing exome studies, which are mainly focused on *de novo* variants and not designed to capture inherited variants^8, 9, 13, 20, 23, 24^. Investigating both *de novo* and inherited variants has become a favored approach in recent next-generation sequencing based ASD studies^10, 11, 14, 16–18^.

Considering the high rate of consanguinity in the Saudi population, a recessive model was implemented under which a total of 19 rare variants were identified in different genes (Table 2).

In addition to recessive variants, we interrogated changes transmitting in a heterozygous manner (autosomal dominant). By only considering rare variants that were transmitted to the proband from one heterozygous parent, 4 rare variants were confirmed in 4 different genes (*APC2*, *AGL*, *NEB* and *SEMG2*). Two of the variants were observed in ASD-9 occurring within a splice site region of *APC2* and *AGL*. One missense variant affecting *NEB* was found in ASD-39 and a nonsense variant within *SEMG2* was found in ASD-66 (Table 2).

### X-linked variants

A strong male bias in ASD has been consistently observed over time and across numerous studies in different populations^35–37^. This provides a strong clue to the potential involvement of sex chromosomes in the etiology of the disorder, which has been supported by the identification of multiple risk loci, particularly on the X-chromosome^10, 16, 17, 19^.

By setting our analysis approach to identify inherited variants in X-chromosome genes, we detected and confirmed a total of 21 rare variants (19 missense, 1 small deletion and 1 splice site change) and each was found in a different gene (Table 2). Of these variants, 4 were found in genes not previously implicated in ASD nor listed in Autism databases (*DDX26B*, *HTATSF1*, *ITIH6 and PLP1*). The remaining variants existed in genes already reported in ASD or other neurological disorders.

### Description of confirmed rare variants per trio

By tailoring our WES analysis pipeline to capture both *de novo* and inherited variants, the analysis retained at least 2 rare variants affecting different genes for most probands. The variants are discussed in detail in the supplementary data and Supplementary Table S4.

### Biological processes over-represented in our study

IPA functional analysis detected significant enrichment for biological processes such as ‘Cell Signaling’, ‘Cell Morphology’ and ‘Cellular Assembly and Organization’ across the entire genes set. As for Diseases and Disorders, the analysis discovered ‘Neurological Disease’, ‘Organismal Injury and Abnormalities’ and ‘Hereditary Disorder’, among other human diseases, to be significantly correlated. Furthermore, ‘Nervous System Development and Function’, ‘Tissue Development’ and ‘Embryonic Development’ were the 3 top ranking functions under the ‘Top Physiological System Development and Function’ category (Supplementary Table S5).

## Discussion

Dissecting the genetic architecture of an otherwise highly complex disorder such as ASD, comprising diverse forms of genetic alterations (from SNVs to chromosomal aberrations affecting numerous genes/loci), requires employing different approaches. Ideal approaches would be those permitting genetic investigation of ASD cases, in which a polygenic model is assumed (or strongly suspected)^7^. Therefore, capturing more classes of genetic variation, especially with the emergence of studies identifying multiple mutations in individual ASD patients of which all are predicted to contribute to the etiology of the disease, has become the preferred approach by many researchers. We, as others^10^, took advantage of NGS to perform comprehensive analysis in parent-child trios designed to detect both *de novo* and transmitted genetic variants. By considering rare variants with predicted damaging effect, we validated variants in 17 of 19 trios (~90%). We could not comment on whether our detection yield is comparable with other studies or not, due to the lack of published ASD trio exome studies of similar design using the same platform. However, Jiang and colleagues conducted a relatively similar study (utilizing an Illumina platform) in which they have reported successfully detecting medically relevant variants in 50% of their ASD trio families using whole-genome sequencing (WGS)^10^.

Missense variants constitute the most frequently encountered type of variation (77%) in this study followed by loss of function variants [nonsense (6%), frameshifts (6%) and splice site (9%)] (Fig. 2A). Of the 19 probands only 3 were found to carry *de novo* events, whereas rare variants in autosomal or X-linked genes were detected in equal number of probands. While most of the previous studies concerned with sporadic autism have focused on assessing the role of *de novo* variants under the hypothesis that such lesions are more likely to confer risk to ASD than inherited events^9, 12, 13, 20, 24, 38^, we, on the contrary, observe enrichment for inherited variants. This is unsurprising given the highly inbred nature of the Saudi population in which common ancestry is suspected even in apparently non-consanguineous unions as parents are often unaware of consanguinity in previous generations. Such observation is common in Middle Eastern and North African populations^39^.

In our study, 32 of the detected variants were observed in genes previously reported in ASD (either in Autism databases or in literature) (Table 1). For instance, *SUMF1* ^40^, *HSPBP1* ^41^, *TRIM9* ^42^, *DUSP3* ^24^ and *BOC* ^43^. WES detected *de novo* variants in three genes (Table 2). One missense variant affecting a schizophrenia susceptibility gene (*PRODH2*)^44, 45^ and the other two were protein-truncating variants. One was identified in *KDM5B*. This gene was previously associated with ASD^8, 9^ and non-syndromic intellectual disability (ID)^46^ whose function is related to cell cycle control and neural cells differentiation^47^. The other was a frameshift insertion of two adenine bases creating a premature stop codon in *MOGS*. This gene encodes an endoplasmic reticulum glucosidase involved in normal immune function^48^.

By applying the autosomal recessive model, we validated variants in 19 genes (Table 2). One missense variant was observed in *MAN1B1*, a gene causally linked to non-syndromic intellectual disability^49, 50^, and another was found in *DNAJC13* previously reported to be mutated in patients with Parkinson’s disease^51, 52^. Two variants were identified in genes with possible, but not confirmed association with mood disorders: *CRY1* ^53–55^ and *NT5DC1* ^56, 57^. Interestingly, patient (ASD-21) harboring *NT5DC1* missense variant displayed symptoms of depression and poor appetite around the age of 5 years. Amongst the autosomal transmitted variants, six were predicted to disrupt a group of genes involved in brain development and/or function namely *TRIM9*, *BOC*, *SSTR3*, *NGF*, *FGF5* and *CELSR2* ^58–65^, two were found in genes related to protein homeostasis (*KCTD21* ^66^ and *HSPBP1* ^67^) and the remaining variants were identified in genes involved in diverse biological process including cell signaling (*DUSP3*)^68^, inflammatory responses (*NLRP2*)^69^, olfaction (*OR6C65*)^70^, maintenance of DNA integrity (*CEP152*)^71^, and stabilization of the extracellular matrix (*ITIH2*)^72^.

The X-linked model revealed 21 different variants (Table 2). For instance a missense variant was detected in *MXRA5*, a gene that was reported to be mutated in three multiplex ASD families^19^. A deletion of 12 nucleotides was detected within exon 2 of *AVPR2*, defects in this gene cause X-linked congenital nephrogenic diabetes insipidus (NDI) and where detected in dizygotic twins with NDI and ID^73^. One putative splice site variant was found in *PDK3*, a gene that encodes an enzyme involved in the shift of energy production site from the mitochondria (oxidative phosphorylation) to the cytoplasm (glycolysis) reported in cancer cells^74^. The same gene was recently reported to be mutated in a single family with Charcot–Marie–Tooth disease^75^. However, the patient (ASD-38) reported here to carry *PDK3* splice site variant is not affected with Charcot-Marie-Tooth disease, nor does he suffer from any type of neuropathy. Moreover, of the variants detected on the X-chromosome, four were located in genes involved in brain function/development (*PLP1* ^76^, *MAOB* ^77^, *USP9X* ^78^, and *FLNA* ^79, 80^) and two were present in genes regulating splicing and/or transcription (*HTATSF1* ^81^ and *GPKOW* ^82, 83^). Additional rare variants were identified in genes involved in: (i) the hydrolysis of angiotensin II (*ACE2*)^84^ or sulfate esters (*IDS*)^85^ and (*ARSH*)^86^; (ii) or in the ubiquitination and proteasomal degradation of creatine kinase-B (*ASB*-*9*)^87^. Furthermore, two missense variants were detected: one in *ATP2B3* and another in *RPS6KA6*. Both genes participate in signal transduction pathways, however only *ATP2B3* was linked to a human disease^88, 89^. The remaining variants were located in genes of largely unknown function such as (*CFAP47*, *ITIH6* and *ZNF630*) or in cancer-associated genes namely; *INTS6L* ^90, 91^ and *SSX3* ^92^.

With regard to the biological functions that might be influenced by the identified genetic changes, we have compared those highlighted by the IPA with the results of the manual literature mining. Cell signaling was among the common biological themes. Typically, categories pertaining to CNS function such as those found by IPA “Nervous System Development and Function” or manual search “Neuronal function/Development” were over-represented. These categories comprised genes serving diverse functions in the CNS such as neurite outgrowth, synaptic function and formation, and neuronal migration (Dataset 1). Aside from the disruption of neuron-specific functions, defects involving basic cellular processes (presented here and elsewhere) represent another commonly encountered theme in ASD, reflecting the clinical and genetic complexity and variability of this condition^1^. It is noteworthy, that several of the detected rare variants were observed in genes without currently recognized functions/roles making it difficult to propose a functional link to ASD, something that might be revealed by future studies.

One of the interesting findings emerging from this study is the identification of two genes (*NLRP2* and *MOGS*) with established roles in the immune system. The former encodes for NLRP2 protein that plays a vital role in astrocytes innate immunity^69^ and was found to be mutated in a rare type of imprinting disorder known as Beckwith-Wiedemann syndrome^93^. The latter gene (*MOGS*), on the other hand, encodes an endoplasmic reticulum glucosidase involved in normal immune function and is causally linked to congenital disorders of glycosylation type II B clinically characterized by developmental and neurological defects^48^. It is worth mentioning that immune dysfunction has been documented in ASD and emerging studies propose a role for the immune system in the pathophysiology of autism. In support of this notion, typical behavior and neuropathological symptoms of human ASD were successfully recapitulated in mice born to immune-activated (infected) mothers^94^.

Another key finding demonstrated here, is the genetic variability not only across trios but within each proband. Defects in genes involved in diverse biological processes were observed in most probands and only few (ASD-40, 43, 58 and 73) that were enriched for variants affecting neuronal function/development. Our findings are in keeping with a growing number of reports suggesting a genetic model of autism whereby cumulative contribution of multiple inherited and *de novo* variants (multiple hit) in different genes (genetic heterogeneity), including genes associated with other neuropsychiatric disorders (pleiotropy) or involved in diverse biological processes beyond synaptic function (molecular diversity), shape the risk to ASD^1, 10, 11, 16, 95^

## Conclusions

Apart from Yu *et al*. exome sequencing study, aimed at identifying inherited SNVs in a cohort of consanguineous/multiplex families with ASD including two Middle Eastern multiplex families (one from Saudi Arabia and one from Kuwait)^17^, the genetic characteristics of ASD in Arab populations remain largely unexplored. This work represents, to the best of our knowledge, a comprehensive exome analysis of trios with ASD from an Arab population. Our primary goal was to identify *de novo* or rare inherited coding variants of potential clinical relevance through applying WES on case-parent trios from singleton families. Among the advantages of implementing this approach over WGS is that it generates fewer results and therefore is relatively less analytically challenging. Although this approach enabled us to generate a prioritized shortlist of potential deleterious variants, association and extensive functional studies are necessary to identify the disease causing ones with more certainty. Also it is important to consider the possibility of missing candidate variants within coding regions and the fact that neither common variants nor non-coding regions were considered here. In spite of the small number of cases investigated here; our current study is useful in terms of contributing a small, albeit rich data set, revealing new ASD candidate genes that may shed light on potential diagnostic and therapeutic targets.

## Methods

### Ethics statement

Patient recruitment and all experimental protocols used in this study were in compliance with the Declaration of Helsinki and were approved by the institution’s relevant committees; Internal Review Board (IRB), Research Ethics committee and Basic Research Committee at King Faisal Specialist Hospital and Research Center. Written informed consent was obtained from subjects before enrollment in an IRB-approved protocol (King Faisal Specialist Hospital and Research Center (KFSHRC) RAC#2080001).

### ASD trios selection

A total of 19 simplex (singleton) families were selected for the study (Trio’s analysis). Venous blood samples from parents and affected child were obtained for both DNA and RNA extraction. Of the recruited families, 9 were from consanguineous marriages and 10 were not. Non-consanguineous families were included based on the rationale that in populations with high levels of consanguinity/endogamy (tribal or religious)^96^, a common lineage is suspected even when couples regard themselves as unrelated as they are often unaware of consanguinity in distant generations^39, 97^. Diagnosis of ASD was based on the Diagnostic and Statistical Manual of Mental Disorders, Fourth Edition, Text Revision (DSM-IV-TR) criteria (American Psychiatric Association [APA], 2013) and ADI-R, ADOS (which are not yet validated in Arabic). Both parents were present for the interviews and assessment. All selected cases did not have symptomatic ASD secondary to known genetic or metabolic disorders (such as Fragile X syndrome, Tuberous Sclerosis, Rett syndrome, Angelman, Prader-Willi syndrome or Phenylketonuria). Available clinical and demographic information is summarized in Supplementary Table S6.

All probands were negative for any copy number variants (CNV) in previously reported ASD-associated loci/genes listed in Supplementary Table S7. CNV analysis was performed in probands using the Cyto Scan HD array (Affymetrix, Santa Clara, CA,USA). Targeted analysis was carried out using the Chromosome Analysis Suite version Cyto 3.0 using GRC 38/hg19 of the UCSC Genome Browser and the recommended threshold of log2 ratios of more than 0.58 for CNV gains and less than -1 for CNV losses was used.

### Whole exome sequencing, data processing and bioinformatics analysis

#### WES, alignment of reads, and variant discovery

The exomes of the parents and affected children were sequenced on the Ion Proton platforms, using the whole exome AmpliSeq kit. Briefly, 12 separate Exome Primer Pools, AmpliSeq HiFi mix (Life Technologies, Carlsbad, CA, USA) and 100 ng DNA from each sample were used in the amplification step for 10 cycles. The resultant PCR products were then pooled in preparation for primer digestion using FuPa reagent (Life Technologies, Carlsbad, CA, USA). This was followed by a ligation step using Ion P1 and Ion Xpress Barcode adapters. The libraries were then purified and quantified using qPCR and the Ion Library Quantification Kit (Life Technologies, Carlsbad, CA, USA) prior to emulsion on an Ion OneTouch System. The templated Ion Sphere particles were enriched using Ion OneTouch ES (Life Technologies, Carlsbad, CA, USA) and processed for sequencing on the Ion Proton instrument (Life Technologies, Carlsbad, CA, USA). Approximately 15–17 Gb of DNA sequence was generated per sequencing run/sample. Reads were mapped to UCSC Human reference genome (hg19) (http://genome.ucsc.edu/) and variants were identified using the Ion Torrent pipeline (Life Technologies, Carlsbad, CA, USA).

The sequencing targets 293903 amplicons, covering about 19,000 genes (https://www.ampliseq.com/tmpl/view.action?tmplDesignId=50055032). The analysis pipeline is a multi-step process (Fig. 1). First, the NGS reads were subjected to quality control checks for removing any low quality reads. Then the reads were mapped (aligned) to hg19. For the mapping step, we used the program tmap, which is part of the Torrent Suite package. This program is the same as BWA but it is tuned more to the Ion Torrent technology, by including flow signal information in the alignment process. Next, variant calling was performed using the Torrent Suite Variant Caller, which is a GATK-like variant caller, but more tuned to the Ion NGS data. It takes flow signals into account and also recognizes types of sequencing errors common to this platform.

As part of a tertiary data analysis, each trio was further interrogated using four possible inheritance models; autosomal recessive, autosomal dominant, *de novo* and X-linked. To that end, we compared the variants detected in the affected child to the corresponding positions in the parents DNA. Basically, we grouped the variants into model-specific subsets according the mode of inheritance. For instance, a variant was considered to be *de novo* if it was present uniquely in the proband and absent in both parents, while if two copies of the variant were inherited by heterozygous parents, the variant was considered to be autosomal recessive (with consideration of compound heterozygosity). On the other hand, the variant was considered autosomal dominant only if it was transmitted to the proband from one heterozygous parent. Moreover, for a variant to be considered as X-linked, it had to occur on the X-chromosome in either a hemizygous (male) or a homozygous (female) state in the proband in reference to the parents.

#### Variants annotation

ANNOVAR (http://annovar.openbioinformatics.org) software was used to functionally annotate detected variants as previously described^98^. Briefly, the software was used to perform two types of functional annotations; gene-based and filter-based. The former one identifies the genomic region containing the variant (e.g exonic or intronic), and the latter assesses the frequency of the variant in widely used databases in addition to providing functional prediction scores from a number of tools.

#### Variants prioritization and Sanger validation

The resulting model-specific set of variants was trimmed by omitting the ones not falling within coding exons or exon/intron boundaries, or non-functional. Then validation by Sanger sequencing was carried out on fresh DNA aliquots prior to assessing the frequency and predicted functional effect of the detected variants. The purpose of this was to avoid overlooking any interesting changes that may have been masked by variant calling/annotation errors. The list of validated variants was further trimmed by omitting those reported in dbSNP, or present in international databases (1000 Genomes project, ExAC) or in the local ethnically matching normal controls database (SHGP 2379 exomes) with a MAF ≥ 1%. Variant deleteriousness was predicted using four different web-based tools: Polyphen-2, SIFT, MutationTaster and CADD as described^99–102^. For predicting splice-site variants effect, we utilized PredictSNP2 web interface for its improved performance compared to CADD^103^. In addition, available public Autism genes/variants databases (SFARI Gene; https://gene.sfari.org/autdb/Welcome.do, and AutismKB; http://autismkb.cbi.pku.edu.cn/) were checked for reported SNVs and CNVs in our list of validated genes or bands harboring these genes in case of CNV^104, 105^. Finally, segregation analysis was carried out in families where DNA from unaffected family members (siblings) was available (ASD-17,-24, -37 and -69).

#### Relatedness assessment

Three different relationship inference algorithms were employed in this study to verify the self-reported pedigree information. The first is Yang *et al*. method, which is based on the *A*_*jk*_ statistics^106^. For this method we used the implementation in the VCFtools package (with option –relatedness 1). In the second method, we computed shared homozygosity by comparing the homozygous variants (MAF > 1%) between each pair of individuals and counting the number of overlapping variants. Relatedness was denoted as “Not confirmed” if the number of shared homozygous variants was less than 25 SD above the mean (123 ± 25). The third method estimates kinship as described by Manichaikul and colleagues^107^ using KING program (http://people.virginia.edu/~wc9c/KING/) and the implementation in VCFtools (option –relatedness2).

### Biological functions analysis

In an attempt to gain insight into the biological processes that may potentially be affected by the variants identified herein, we analyzed our final list of genes with validated variants using the core analysis function of Ingenuity Pathway Analysis software (IPA) (IPA®, version 01–07, QIAGEN, Redwood City, https://analysis.ingenuity.com/pa/installer/select). The software identifies the diseases and biological functions that are significantly associated with our genes list. The resulting categories are ranked by p-values calculated using right-tailed Fisher’s exact test.

In parallel, we have grouped gene-sets on the basis of their function (by means of manual literature research) into different categories including: “Neuronal function/Development”, “Mitochondrial function/Energy metabolism”, “Protein quality control”, “Gene regulation”, “Cell signaling cascade”, “Cell division or differentiation”, and “Immune responses” (Table 1).

## Electronic supplementary material

Supplementary information

Dataset1
