# Supplementary material for: Whole exome sequencing reveals inherited and de novo variants in autism spectrum disorder: a trio study from Saudi families
Source: Sci Rep. 2017 Jul 18;7:5679. doi: 10.1038/s41598-017-06033-1 (PMC5515956; doi:10.1038/s41598-017-06033-1)
Supplement: Supplementary file 1 — Supplementary information [file 41598_2017_6033_MOESM1_ESM.pdf]

Supplementary Information

Whole exome sequencing reveals inherited and *de novo* variants in autism spectrum disorder: a trio study from Saudi families

Bashayer Al-Mubarak[1,2*], Mohamed Abouelhoda[2,3], Aisha Omar[1], Hesham AlDhalaan[4,5], Mohammed Aldosari[4], Michael Nester[5], Hussain A. Alshamrani [6], Mohamed El-Kalioby[2,3], Ewa Goljan[2,3], Renad Albar[2], Shazia Subhani[2,3], Asma Tahir[1], Sultana Asfahani[4], Alaa Eskandrani[4], Ahmed Almusaiab[1], Amna Magrashi[1], Jameela Shinwari[1], Dorota Monies[2,3], Nada Al Tassan[1,2*].

[1] Behavioral Genetics unit, Department of Genetics, King Faisal Specialist Hospital and Research Center. P.O Box 3354, Riyadh 11211, Saudi Arabia

[2]Saudi Human Genome Program, King Abdulaziz City for Science and Technology, Riyadh, Saudi Arabia

[3] Department of Genetics, King Faisal Specialist Hospital & Research Centre. P.O. Box 3354, Riyadh 11211, Saudi Arabia

[4]Center for Autism Research, King Faisal Specialist Hospital & Research Centre.P.O. Box 3354, Riyadh 11211, Saudi Arabia

[5]Department of Neurosciences, King Faisal Specialist Hospital & Research Centre. P.O. Box 3354, Riyadh 11211, Saudi Arabia

[6] Pediatric Department, King Faisal Specialist Hospital & Research Centre. P.O. Box 3354, Riyadh 11211, Saudi Arabia

[*]**Corresponding authors: naltassan@kfshrc.edu.sa (NAT)** and **BAl-Mubarak@kfshrc.edu.sa (BA)**

Description of confirmed variants per trio

ASD-9

Our analysis revealed two variants in this patient that were detected when the autosomal dominant model was applied. Both variants affected a splicing site, one (c.294-2A>T) in *AGL* and the other (c.1207+3G>C) in *ACP2*. Exons flanking the affected splice sites in both genes were present in all protein coding transcripts. The former gene *AGL* encodes the glycogen debranching enzyme (GDE). This enzyme is necessary for glycogen metabolism in organs such as the liver and skeletal muscles and deficiencies in which, result in incomplete glycogenolysis. Autosomal recessive mutations in *AGL* have been linked to glycogen storage disease type III (GSD III), but to the best of our knowledge, none have been reported in patients with neurological conditions (Goldstein, et al., 2010). However, ample experimental evidence for the importance of glycogenolysis in learning and memory exist. The evidence comes from studies demonstrating a key role for glycogen-derived lactate (released from astrocytes and taken up by neurons) in normal brain function. This concept is substantiated by memory impairment observed upon glycogenolysis inhibition that could be rescued by lactate (Hertz, et al., 2013).

As for *APC2*, studies in rat neuroblastoma and chick embryos have shown the product of this gene to be an essential player in the development of axonal projections [1]. More recently, a homozygous mutation was identified in two siblings diagnosed with Sotos syndrome characterized by sever neurological features such as intellectual disability and abnormal brain structure. Moreover, *Apc2* deficient mice recapitulated the phenotypes observed in patients with Sotos syndrome and highlights the importance of APC2 for normal brain development and function [2]. Of note, no indication for GSD III or Sotos syndrome diagnosis was found in the available clinical information on this patient.

ASD-16

The proband (a female) had 5 missense variants with a predicted protein-altering effect and a deletion of 12 nucleotides, three of which were transmitted by both parents. The first one (p.V633F) disrupts *MAN1B1*, a gene known to cause non-syndromic intellectual disability [3,4] and has recently been identified as a culprit gene in a number of unsolved cases with congenital disorders of glycosylation type II, displaying global developmental delay and intellectual disability [5,6]. Of note, no indication for glycosylation type II or other metabolic disorders was found in the available clinical information on this patient. The gene product, mannosidase alpha class 1B member 1 (MAN1B1), was initially suspected to reside in the endoplasmic reticulum (ER) and to function in ER-associated degradation (ERAD) on the basis of; 1) the functional and localization information about its yeast orthologue (Mns1p), 2) the localization of exogenously expressed MAN1B1 in mammalian cells to the ER [5]. However, later reports on endogenous MAN1B1 localization to the Golgi-complex in several human cell lines uncovered its contribution in the Golgi-based protein quality control as part of a backup system for recycling misfolded proteins that have escaped the ERAD surveillance back into the ER [7].

The second missense variant (p.R51H) was detected in *KCTD21*, encoding KCTD21 a member of a family of proteins that share a conserved (BTB) domain with voltage-gated K^+ channels and are involved in various biological processes including ubiquitination, gene expression regulation, cell proliferation and apoptosis [8]. One well-characterized role of KCTD21, is as an adaptor molecule for Cul3 ubiquitin ligase mediating the

ubiquitination and subsequent degradation of substrate proteins specifically HDAC1 (commonly activated in cancer cells). Silencing and allelic loss of *KCTD21* have been observed in human medulloblastoma and are believed to lead to tumor formation by preventing HDAC1 degradation [9]. With respect to neurological disorders, mutations in other members of the KCTD family namely, *KCTD7* and *KCTD13* have been linked to progressive myoclonic epilepsy and abnormal head size, respectively [8]. However, mutations in *KCTD21* have not yet been implicated in neurological disorders, with the exception of a single genome-wide association study identifying an intronic SNP within *KCTD21* among a list of top 100 SNPs associated with Schizophrenia [10].

The third homozygous missense variant (p.V147I) occurred in *DUSP3*, an atypical dual specificity phosphatase that is thought to regulate the MAPK cascade components with debatable substrate specificity [11] and was found to play an essential role in angiogenesis [12]. MAPKs such as ERK1/2 and JNK operate in signaling pathways critical for determining cell fate, function and development of neurons within the CNS [13,14]. Besides its role in normal physiology, aberrant DUSP3 expression was observed in different types of cancers [11]. More importantly a missense variant was detected in this gene in one case with ASD[15].

The remaining variants occurred in X-linked genes, two of which were detected in genes with an as-yet-unknown function (p.M2981I in *CFAP47* and p.R90S in *SSX3*) and therefore their role in the disease remains unclear [16]. In addition, a paternally inherited heterozygous deletion of 12 nucleotides (c.739-750 delCGCCGCAGGGGA) was found in *AVPR2*, the product of which is a receptor for the neuromodulator (arginine vasopressin/antidiuretic hormone) that has a key role in controlling social behavior in mammals [17,18] and in urine production [19]. Mutations in *AVPR2*, are the primary cause of NDI. While there is not enough evidence linking *AVPR2* to mental disorders, save for a single report of an X-chromosome deletion encompassing the entire *AVPR2* gene in monozygotic twins with NDI and ID [17], evidence exist for the possible contribution of specific genetic variants in the other receptor subtype (*AVPR1A*) to risk for ASD [18].

ASD 17

Two missense and one nonsense variant was detected in this male proband. The nonsense (p.W91X) variant seen in *CRY1* and the missense variant (p.W175R) in *NLRP2*, both appear to be inherited from both parents. The premature stop codon in the former gene appears to have escaped non-sense mediated mRNA decay (as demonstrated by the presence of a stable transcript from the proband (Supplementary Fig. S2)) and therefore, it is predicted to produce a truncated form of the protein. Despite the high recurrence of sleep difficulties in children with ASD [20] and the physiological role of *CRY1* in sleep regulation [21] , a link between *CRY1* genetic variation and ASD has not been previously suspected. However, no reports of sleeping difficulties were documented in the available clinical data for the proband in question. The latter gene product NLRP2, along with other members of the same subfamily (pyrin domain-containing NLR proteins), have been shown to modulate inflammatory responses via inhibition of NF-κB [22]. As for the CNS, Minkiewicz and colleagues, have provided evidence for NLRP2 expression in human astrocytes as part of the NLRP2 inflammasome multiprotein complex which is a main component of the glial innate response to CNS injury [23]. To date only a single human disease was associated with defective *NLRP2*. The disease is a rare familial form of Beckwith-Wiedemann syndrome, a type of human imprinting disorder, with complex genetic etiology involving a number of genes with critical roles in early development [24].

ACE2 is another gene that was found to be affected by a maternally inherited missense variant (p.A412E) in proband ASD17, which encodes angiotensin converting enzyme (ACE2). Of note, the same variant was

observed in the unaffected sibling . This enzyme operates in the brain renin angiotensin system by degrading angiotensin II to shorter peptides (Angiotensins I-VII). The effect of the intact angiotensin II is mediated by two types of angiotensin receptors (I and II). Activation of angiotensin receptor II induces key signaling pathways pertaining to brain development and cognition [25]. However, the actions of angiotensin II are counteracted by angiotensins (I-VII). Moreover, most of the reports on *ACE2* role in pathophysiology come from studies investigating the contribution of gene polymorphisms to risk of cardiovascular diseases, with inconsistent results [26].

ASD 18

This male patient harbored 5 variants including 4 were inherited in an autosomal recessive manner and one as an X-linked. A homozygous deletion at the splice site of *GLT8D1* (c.811_812+2del GAGT) was observed. This deletion introduced an amino acid substitution (p.E271K) as revealed by Sanger sequencing analysis of the cDNA (Supplementary Fig. S1A). The functional consequence of this variant and how it may contribute to ASD is hard to predict given that the gene function is not fully characterized. However, a single study in Japanese patients with schizophrenia or major depressive disorder (MDD) indicated a significant correlation of a particular intronic SNP within *ITHI3*, previously associated with a number of psychiatric disorders including ASD, with increased levels of GLT8D1 in MDD patients [27]. Until these results are corroborated by independent studies and replicated in ASD patients, the role of *GLT8D1* remains to be elucidated.

Another homozygous variant was present in a gene (*DNAJC13;* p.N1865S) formerly associated with PD [28]. The gene product is a component of the endosomal protein sorting machinery which together with lysosomal proteins form the endosomal-lysosomal system involved in membrane proteins sorting, trafficking and recycling and has recently emerged as a defective pathway in ASD and a host of neurodegenerative disorders[29] [30].

A second missense variant, inherited from both parents, was observed in *HSPBP1*, a member of the Hsp-70 co-chaperone family that facilitate folding of nascent proteins, restoration of damaged protein conformation and degradation of client proteins. Under stressful conditions when proteostasis is in high demand, inducible members including HSPBP1 are activated to enable the cells to cope [31,32]. Like many other biological processes, proteostasis declines with aging and in disease states for instance diabetes II, cancer and neurodegenerative diseases [33]. With regard to psychiatric disorders, it is noteworthy that *HSPBP1* was among a list of dysregulated genes shared by autism with FMR1-FM and with dup (15q) [34].

We also detected a frameshift change (p.M256fs) in a member of a large family of olfactory receptors (*OR6C65*) [35]. While there are no prior reports of *OR6C65* genetic alteration in ASD patients, dysregulated expression of a related olfactory receptor (*OR2L13*) was observed in blood, postmortem brain, and buccal epithelial samples of ASD cases [36]. Moreover, abnormal olfaction, be it enhanced sensitivity or inappropriate sniffing responses have been documented in ASD cases [37,38].

A single variant (p.P560A) was found in *ATP2B3* on chromosome X. The gene encodes plasma membrane calcium pump/plasma membrane Ca^{2+} ATPase isoform 3 (PMCA3) , alternatively known as ATP2B3, which operates in concert with other plasma membrane and organelle membrane located pumps to maintain Ca^{2+} homeostasis [39]. PMCA3 and PMCA2 are predominantly (albeit not exclusively) expressed in the brain and their essential role in normal central nervous system function is highlighted by the phenotypes induced by genetic manipulation of the latter, for instance deafness and ataxic features observed in mice lacking this gene. On the

other hand, *PMCA3* knock out animals have not been developed yet, most likely due to embryonic lethality, however, a missense mutation leading to impaired pump function was identified in a family with X-linked congenital cerebellar ataxia. In addition, microduplication at the long arm of chromosome X comprising *ATP2B3*/*PMCA3*, of unknown clinical significance, gene was detected in a single case with ID [40].

ASD-19

Two missense variants in X-linked genes and one *de novo* nonsense variant were identified in this male proband. One of the missense variants (p.S385G in *ITIH6*) occurred in a functionally non-annotated gene (*ITHI6*) [41]. The other missense variant (p.L10F) existed in a gene (*MXRA5*) that was previously found to harbor rare possibly deleterious variants in three unrelated multiplex families with ASD [42]. MXRA5 is a proteoglycan belonging to the extracellular matrix (ECM) remodeling and cell-cell adhesion group of glycoproteins. Although the specific function of MXRA5 is unknown, ECM glycoproteins serve a number of roles in the developing and adult nervous system, for instance, regulation of neural stem cells behavior, axonal growth, myelination and neuronal synaptic function [43].

The *de novo* nonsense variant (p.Y755X) resides in *KDM5B* encoding for Lysine-specific histone demethylase 5B, whose functions include maintenance of genome stability, and control of cell cycle, differentiation and linage genes [44,45]. Close homologues of this gene *KDM5A* and *KDM5C* have been implicated in the autosomal recessive and syndromic X-linked forms of ID, respectively [3,46] and shortly after, a *de novo* splicing mutation within *KDM5B* was identified in a mild case of non-syndromic ID [47]. More importantly, *KDM5B* was among 27 genes harboring recurrent coding *de novo* mutations reported by Iossifov *et. al.* in a subset of Simon simplex collection families [48].

ASD-21

Only two variants were identified in this proband, both transmitting in an autosomal recessive manner. A missense variant (p.S372P) was detected in *NT5DC1*, a gene encoding a protein of unknown function. However, SNPs within this gene were previously associated with bipolar disorder [49,50]. It is noteworthy that the proband displayed symptoms of depression and poor appetite around the age of 5 years. The second missense variant (p.R548H) was found in *TRIM9*, a gene formerly identified as a putative disease causing gene in a whole exome study conducted on 30 Caucasian females with ASD [51]. The protein encoded by this gene (TRIM9) functions as an E3 ubiqutin ligase expressed in murine and human brains that is required for Netrin1-dependent axonal branching control [52]. It is noteworthy that axonal branching aberration has been reported in neurodevelopmental disorders including autism [53-55]. Besides neurodevelopmental disorders, repressed TRIM9 immunoreactivity was observed in post mortem brain sections from patients with PD or dementia with Lewy bodies compared to their normal counterparts and was found to co-localize with α-synuclein in the diseased brains. However, based on its function as an E3 ligase ubiquitin, its co-localization with α-synuclein, and the predominant expression in the neuronal cytoplasm and proximal dendrites, it is reasonable to suggest that TRIM9 in the examined brains with neurodgenerative disorders is more likely to be involved in the formation or elimination of Lewy bodies rather than axonal branching [56].

ASD-24

Two of the three variants observed in this proband were present in X-linked genes. One (p.F298L) was identified in *HTATSF1*, encoding a protein that was initially identified as a co-factor for HIV-1 gene transcription and was later found to take part in the transcription and splicing of cellular genes some of which are involved in cell cycle or nucleic acid metabolism [57]. The second variant (p.A161V) occurred in *GPKOW* which encodes a nuclear RNA-binding protein required for human pre-mRNA splicing [58]. However, their contributory role is undermined since the proband's unaffected sibling was found to carry the same variant. The third variant was a small *de novo* deletion affecting *MOGS* that is predicted to create a premature termination codon (p.K278Efs*5). This gene encodes an endoplasmic reticulum glucosidase involved in normal immune function and is causally linked to congenital disorders of glycosylation type II B clinically characterized by developmental and neurological defects [59]. Of note, no indication for glycosylation type II or other metabolic disorders was found in the available clinical information on this patient . Interestingly, this is the second proband identified here with a variant in a gene associated with congenital disorders of glycosylation type II. The first (ASD-16) harbored a missense variant in *MAN1B1* as described above.

ASD-37

This male proband possessed only a single variant (p.D175E) occurring in an X-chromosome located gene, *IDS*. The gene product, idurunate-2-sulfatase, functions in the lysosomal degradation of glycosaminoglycans (dermatan and heparin sulfates). Deficits in this enzyme lead to the progressive accumulation of its catabolic substrates in multiple organs including the brain causing a type of lysosomal storage disease known as mucopolysaccharidosis type II (MPSII/ /Hunter disease). The severities of the phenotypes associated with this disease vary, from a mild course to a more severe form with early onset, cognitive dysfunction, skeletal deformities and short life expectancy. Although genetic defects in *IDS* have not yet been linked to ASD, the developmental delay and neurological deterioration characteristic of the severe form of MPSII points to a possible role for *IDS* in neurodevelopment [60].

ASD-38

A total of 3 variants, all existing in X-linked genes, were found in this patient. One splice site variant was observed in *PDK3* which encodes a brain expressed isoenzyme involved in energy-production regulation that was found to be mutated in a large kindred with Charcot-Marie-Tooth disease [61]. This splice site change (c.248+3A>G) within *PDK3*, is less likely to be causal as it does not alter the transcript splicing pattern nor the sequence, however, the possibility of the variant being leaky cannot be ruled out as normal transcript abundance has not been assessed (Supplementary Fig. S1B). The other two variants were both missense: one (p.T104P) was detected in *ASB-9*, and the second (p.G110S) in *PLP1*. The biological function of *ASB9* product remains largely undefined save for a couple of studies demonstrating a role for ASB9 in regulating the protein level of cytosolic creatine kinase B (CKB) by acting as an E3- ubiquitin ligase. Interestingly, CKB, predominantly expressed in the brain and the retina, functions in the creatine kinase system critical for cellular energy metabolism in active tissue with high and rapidly changing energy requirements such as the brain and muscles. Moreover, perturbation in the creatine kinase system has been implicated in human pathologies involving the kidney, brain and muscles [62]. Furthermore, reduced habituation and slow spatial learning acquisition was observed in mice lacking *CKB* [63], this phenotype was exacerbated when both brain-type isoforms of creatine kinase (CKB and mitochondrial ubiquitous creatine) were simultaneously ablated [64]. Both studies support a role

for CKB in brain function. As for *PLP1*, this gene produces a major constituent of the CNS myelin (proteolipid protein 1), and is causally linked to two types of myelinopathies; Pelizaeus-Merzbacher disease and spastic paraplegia type 2 [65]. Proper myelination is an important prerequisite for fast and efficient propagation of neuronal impulses that when defective can lead not only to neurological disorders affecting motor function and development, but also, can lead to a range of psychiatric illnesses such as depression and schizophrenia [66]

ASD-39

In this male proband, none of the variants detected by the main three inheritance models (*de novo*, autosomal recessive or X-linked) survived our filtering criteria except one variant (p.L1980S) that was revealed in *NEB* when the autosomal dominant model was implemented. The product of this gene (Nebulin) is involved in actin regulation and is abundantly expressed in skeletal muscles, however, different isoforms of this protein are expressed in other organs including the brain, in which the biological role of nebulin is not yet determined [67]. Three different forms of myopathies (congenital, distal and core-rod) are known result from autosomal recessive mutations in *NEB* [68,69]. Recently, compound heterozygous variants were reported as possible disease-causing mutations in two siblings of a Korean descent with intellectual disability, epilepsy and congenital myopathy. Although, Jin *et al.* point out the need for functional studies to confirm the effects of these variants on the CNS, a role of NEB in normal brain function remains plausible. This could be inferred on the basis of NEB involvement in actin regulation, as dysfunctional actin cytoskeleton can cause abnormal morphological and functional changes of neurons [70].

ASD-40

This patient had only two variants, both transmitted in an autosomal recessive manner in genes with functions related to neuronal synaptic development. One variant (p.G542R) occurred in *BOC*, a gene that encodes a cell adhesion molecule that acts as a noncanonical receptor for Sonic Hedghog (Shh). During cortical development, Boc/Shh signaling was found to regulate synaptogenesis in a layer-specific manner [71]. Although evidence suggesting *BOC* association with ASD is currently lacking, it has been recently shown to be among the genes encompassed by the 3q13.31 microdeletion syndrome in a patient with additional clinical features including autism [72]. In addition, muscular hypotonia which is one of the main symptoms of 3q13.31 microdeletion syndrome, was reported in this patient. Similarly, the second variant (p.A242P) was found in a gene (*SSTR3*) implicated in synapse formation. It encodes the G protein-coupled receptor somatostatin receptor 3, which is among a number of signaling molecules enriched in neuronal cilia with an important role in synaptic function demonstrated by *in vitro* studies reporting disrupted dentritic arborization in cortical neurons overexpressing Srrt3 and by knockout mouse models displaying learning deficits [73,74].

ASD-43

In this patient, we have identified three missense variants all inherited in an autosomal recessive manner. One (p.Q237R) was found in *SUMF1*, a gene responsible for the lysosomal storage disease multiple sulfatase deficiency. Through enhancing the activity of steroid sulfatases, a group of enzymes involved in the metabolism of neurosteriods, SUMF1 protein may indirectly modulate neuronal excitability [75,76]. Moreover, CNVs disrupting this gene have been previously reported in autism cases [77,78]. The third variant (p.R121W) was found within *NGF*. Mutations in this gene have been identified in patients with hereditary sensory and autonomic neuropathy type V. This rare form of the disease is characterized by impaired pain and temperature sensation, orthopaedic symptoms and in some cases delayed mental development [79]. Neurotrophic factors such as the

nerve growth factor, encoded by *NGF*, and the brain-derived growth factor, both play a fundamental role in shaping brain connections during development and into adulthood [80]. A role for neurotrophic factors in the etiology of ASD could be postulated on the basis of their role in modulating brain function and the frequently reported disruption of brain connectivity in autistic patients [81].

ASD 58

A total of 3 variants were found in this patient: 2 in X-linked genes and 1 *de novo*. One of the X-linked genes altered in this patient (*ZNF630*) is of unknown function, while the second (*MAOB* harboring p.S131I substitution) encodes monoamine oxidase B that catalyzes the deamination of a number of neurotransmitters including serotonin and dopamine. Association between specific SNPs within *MAOB* and susceptibility to schizophrenia was suggested in some studies on Chinese, Spanish and Mexican patients [82-84], but none exist with regard to ASD apart from two studies reporting unchanged MAOB plasma levels and activity in Omani and Egyptian autistic children, respectively, compared to their normal counterparts [85] (Essa *et al.*, 2011). However, mice lacking either MAOA, the other brain-expressed isoform, or both MAOA/B display autistic-like behavioral abnormalities [86]. The single *de novo* change (p.G206R) observed in this patient occurred in *PRODH2*, whose product is required for normal synaptic function [87]. Also genetic variations in this gene have been associated with increased susceptibility to schizophrenia [88].

ASD 64

All three variants identified in this patient occurred in X-linked genes including one with unidentified function (*DDX26B*). The other two X-linked genes (*USP9X* and *RPS6KA6*) were previously implicated in X-linked ID. *USP9X*, harboring p.Y1268C substitution, encodes a deubiquitylating enzyme involved in regulating several aspects of CNS development such as neuronal migration, axonal growth and neural progenitor cells fate [89,90]. Exome analysis of the X-chromosome revealed *USP9X* as a high-ranking gene harboring potentially pathogenic variants in 5 individuals with non-syndromic ID, two of which displayed autistic behaviors [89]. Limited information is available on the biological function of RSK4 encoded by *RPS6KA6*, however, a possible but not yet confirmed association with non-specific ID was reported. Moreover, ablation of this gene resulted in learning and developmental defects in Drosophila and mouse, respectively [91].

ASD 66

None of the variants detected in the DNA of this patient by three analysis models survived our filtering criteria. We therefore applied the autosomal dominant model and found one nonsense variant within in *SEMG2*. Semenogelin II, the protein encoded by *SEMG2*, is a main constituent of human seminal plasma that controls sperm motility and capacitation and can influence fertility when dysregulated. The expression of this protein was previously thought to be restricted to the seminal vesicles, however, Lundwall *et al.* detected expression in other tissues including the brain. The presence of semenogelin in a variety of tissue may indicate that it has functions beyond those pertaining sperm physiology or fertility [92]. It is noteworthy that semenogelin II fragments were detected in the cell surface adhesion complexes of small-cell lung carcinoma. More interestingly, small-cell lung carcinomas are derived from the neural crest of the developing fetal brain, however, whether or not semenogelin II has a role in the development of CNS is yet to be discovered [93,94].

ASD 69

All the variants detected in this male patient transmitted in an autosomal recessive manner, except one (p.I375T) occurring in an X-linked gene, *ARSH*, that has been observed in an unaffected male sibling. The two missense variants detected in this patient were (p.D2729H) in *CELSR2* and (p.D573A) in *CEP152*. Knockout experiments in mice have shown that the cadherins *Celsr2* and *Celsr3* act redundantly to each other and in collaboration with a third molecule *Fzd3* in regulating axonal guidance within the forebrain [95]. In addition to axon development, *Celsr2* may be involved in dendritic growth and stabilization as demonstrated by knockdown studies in rat brain slice cultures [96]. On the other hand, *CEP152*, whose gene product has been identified as a regulator of genomic integrity, is not directly linked to brain development or function. However, it has been found to be mutated in patients presenting with primary microcephaly and Seckel syndrome displaying different degrees of cognitive impairment [97]. A small insertion of four nucleotides, that is predicted to result in a frameshift introducing a premature termination codon (p.V622Yfs*17), was found in *ITIH2*, a gene belonging to a family of plasma protease inhibitors dysregulated in multiple human solid tumors. There is currently no data linking *ITIH2* to ASD or even brain function, however, a single polymorphism within *ITIH3*, another gene from the same family, has been associated with increased risk to psychiatric disorders including ASD. Whether genetic variants in *ITIH2* confer risk or protection to ASD has yet to be investigated.

ASD 73

A total of three variants have been found in this patient, one transmitting in an autosomal recessive manner and the rest were present in X-linked genes. All of the mutated genes have functions related to neuronal development or function. For instance, *FGF5* harboring homozygous p.S84L substitution encodes fibroblast growth factor-5, a protein that is involved in promoting differentiation of cholinergic and sterotonergic central neurons as well as the differentiation of midbrain astrocytes [98,99]. Another gene is *FLNA*, harboring (p.V2360A), belongs to the filamins family of actin-binding proteins. Recently, elevated FLNA levels were observed in tuberous sclerosis mouse models and patients, and have been suggested to contribute to the abnormal dendtritic morphology, which is a shared feature of many neurodevelopmental disorders [100]. The third gene *SMS*, harboring (p.L142F), encodes spermine synthase which converts one type of polyamine (spermidine) to another (spermine). Endogenous spermine has been shown to modulate the function of N-methyl-d-aspartate (NMDA) subtype of glutamate receptors, which are critical for learning and memory and are often implicated in neurodegenerative and neuropsychiatric disorders [101]. More importantly, mutated *SMS* has been identified in patients with X-linked intellectual disability [102].

Supplementary Tables

Supplementary Table S1. Sequencing depth, coverage and other metrics for exomes of the probands (data for parents' exomes are similar and not shown).

Proband-ID	Mean Read Length at Q0	Mean Read Length at Q20	Reads at Q0	Reads at Q20	Average Depth	Target base coverage at 1x	Target base coverage at 20x
ASD-19	169	100	84556203	84556203	226.2	99.21%	95.70%
ASD-18	165	115	67765247	67765247	201.4	98.70%	95.10%
ASD-17	167	110	85664171	85664171	224.8	98.82%	94.73%
ASD-39	164	97	76700001	76700001	199.7	98.86%	95.14%
ASD-40	165	93	89732941	89732941	231.9	99.23%	93.23%
ASD-43	165	96	85894164	85894164	223.9	98.74%	93.93%
ASD-52	173	123	83256475	83256475	227.6	99.29%	95.93%
ASD-58	163	87	90164698	90164698	228.2	99.13%	95.55%
ASD-64	154	65	81749965	81749965	196.7	98.61%	93.87%
ASD-66	173	114	86658907	86658907	239.3	98.71%	94.97%
ASD-69	173	120	96264954	96264954	261.3	98.99%	95.48%
ASD-21	158	63	85801701	85801701	209.7	98.97%	94.62%
ASD-24	176	135	89775465	89775465	253.7	98.51%	94.29%
ASD-37	156	110	85910021	85910021	209.3	98.77%	94.40%
ASD-9	168	122	74026449	74026449	198.9	98.56%	93.97%
ASD-38	164	113	86449490	86449490	219.6	98.83%	94.41%
ASD-16	163	89	84246937	84246937	217.1	98.77%	94.51%
ASD-55	167	108	89117407	89117407	234.9	99.07%	95.43%
ASD-73	171	121	88997986	88997986	239.9	98.92%	95.76%
					**Average=223.37**	**Average=98.81%**	**Average=95.43%**

Q0: without applying read quality step or as it was given by the sequencer; **Q20:** after applying the quality step; **ASD-ID:** Autism spectrum disorder project sample ID. **Q20** was selected to assure confidence in the base call and achieve high quality mapping and variant calling. Coverage at depth 1X and 20X is given in the last two columns.

Supplementary Table S2. Summary of all the detected variants before applying the filtering pipeline.

Sample-ID	Total variant count	Total SNP count	Total INDEL count
ASD-19-P	26123	25187	936
ASD-19-F	29819	28130	1689
ASD-19-M	25470	24629	841
ASD-9-F	28456	27085	1371
ASD-9-M	29042	27592	1450
ASD-9-P	26269	25216	1053
ASD-18-F	25365	24619	746
ASD-18-M	29490	27959	1531
ASD-18-P	24832	24133	699
ASD-16-F	26347	25555	792
ASD-16-M	27255	26124	1131
ASD-16-P	25774	24895	879
ASD-17-F	28136	26676	1460
ASD-17-M	26885	26034	851
ASD-17-P	27781	26384	1397
ASD-39-F	25545	24707	838
ASD-39-M	27746	26231	1515
ASD-39-P	30485	28746	1739
ASD-40-F	31548	29510	2038
ASD-40-M	31670	29808	1862
ASD-40-P	32662	30547	2115
ASD-43-F	27598	26443	1155
ASD-43-M	27133	25954	1179
ASD-43-P	29207	27263	1944
ASD-52-F	32301	30203	2098
ASD-52-M	27876	26460	1416
ASD-52-P	28115	26868	1247
ASD-58-F	28452	27038	1414
ASD-58-M	27404	26207	1197
ASD-58-P	31211	29235	1976
ASD-64-F	29339	27710	1629
ASD-64-M	29917	28124	1793
ASD-64-P	33749	30656	3093
ASD-66-M	29914	28175	1739
ASD-66-P	27929	26530	1399
ASD-66-F	28908	27289	1619
ASD-73-F	30224	28358	1866
ASD-73-M	30837	29020	1817
ASD-73-P	28020	26740	1280
ASD-69-F	27731	26532	1199
ASD-69-M	33564	31308	2256
ASD-69-P	29265	27640	1625
ASD-55-F	30604	27461	3143
ASD-55-M	27894	26517	1377
ASD-55-P	29372	27699	1673
ASD-21-F	35308	30992	4316
ASD-21-M	33519	29529	3990
ASD-21-P	34884	31033	3851
ASD-24-F	29071	27562	1509
ASD-24-M	31592	29479	2113

ASD-24-P	26786	25623	1163
ASD-37-F	26844	25658	1186
ASD-37-M	29912	28028	1884
ASD-37-P	28509	27217	1292
ASD-38-F	29324	27782	1542
ASD-38-M	31154	29318	1836
ASD-38-P	29692	28190	1502
**Average**	**29120.333**	**27466.807**	**1653.526**

F: Father, M: Mother, P: Proband

Supplementary Table S3. Kinship and relatedness assessment.

Family	Member1	Member2	Shared Homozygosity	%Shared Homozygosity	Homo Member1	Homo Member2	A_{jk}_statistics	Kinship	Kinship2	Relation
ASD-19	AS	UF	429	23.4746922	1687	1968	0.287115	0.3786	0.394767	P-C
ASD-19	AS	UM	385	23.6196319	1687	1573	0.313224	0.3813	0.395429	P-C
ASD-19	UF	UM	141	7.963852019	1968	1573	-0.0252781	0.3461	0.319101	NC
ASD-18	UF	UM	143	8.808130582	1508	1739	0.0298864	0.3594	0.32167	NC
ASD-18	UF	AS	484	29.82131855	1508	1738	0.270053	0.3894	0.386441	P-C
ASD-18	UM	AS	358	20.59246477	1739	1738	0.282205	0.3799	0.395951	P-C
ASD-17	UF	UM	157	8.406961178	1721	2014	-0.0154254	0.3447	0.312158	C_L
ASD-17	UF	AS	370	21.87407626	1721	1662	0.316441	0.3447	0.413377	P-C
ASD-17	UM	AS	459	24.97279652	2014	1662	0.286569	0.3761	0.377049	P-C
ASD-39	UF	UM	186	11.10447761	1626	1724	0.0116758	0.3536	0.317873	C_L
ASD-39	UF	AS	424	21.41414141	1626	2334	0.237056	0.3765	0.386047	P-C
ASD-39	UM	AS	453	22.3262691	1724	2334	0.277023	0.389	0.395022	P-C
ASD-40	UF	UM	227	12.07446809	1819	1941	0.0149941	0.3713	0.315678	C
ASD-40	UF	AS	521	23.56399819	1819	2603	0.402962	0.3917	0.39327	P-C
ASD-40	UM	AS	537	23.63556338	1941	2603	0.364779	0.397	0.392016	P-C
ASD-43	UF	UM	144	9.62888666	1485	1506	-0.00524976	0.3559	0.304688	NC
ASD-43	UF	AS	383	23.84806974	1485	1727	0.297014	0.3824	0.377562	P-C
ASD-43	UM	AS	362	22.39406124	1506	1727	0.240124	0.3845	0.377445	P-C
ASD-52	UF	UM	124	6.316861946	2216	1710	-0.0328867	0.3514	0.309336	NC
ASD-52	UF	AS	352	18.78836402	2216	1531	0.287442	0.384	0.388636	P-C
ASD-52	UM	AS	298	18.38938599	1710	1531	0.285713	0.382	0.394707	P-C
ASD-58	UF	UM	122	7.144948755	1582	1833	0.00651877	0.3415	0.32277	NC
ASD-58	UF	AS	337	19.15316851	1582	1937	0.311193	0.3415	0.409545	P-C
ASD-58	UM	AS	391	20.74270557	1833	1937	0.357165	0.3744	0.39322	P-C
ASD-64	UF	UM	129	7.701492537	1620	1730	-0.00848461	0.3506	0.318729	NC
ASD-64	UF	AD	408	21.66179984	1620	2147	0.263592	0.3929	0.389554	P-C
ASD-64	UM	AD	409	21.09878772	1730	2147	0.272818	0.3864	0.388172	P-C
ASD-66	UM	AS	431	23.97774687	2033	1562	0.249268	0.3825	0.383725	P-C
ASD-66	UM	UF	156	8.418780356	2033	1673	-0.0305478	0.3491	0.313749	C_L
ASD-66	AS	UF	357	22.07109737	1562	1673	0.227463	0.383	0.376038	P-C
ASD-69	UF	UM	302	15.73326387	1994	1845	0.0791648	0.355	0.326019	C
ASD-69	UF	AS	692	32.76515152	1994	2230	0.371303	0.3797	0.40226	P-C

ASD-69	UM	AS	609	29.88957055	1845	2230	0.758111	0.3924	0.395962	P-C
ASD-21	UF	UM	735	22.06544581	3036	3626	0.772215	0.3628	0.327678	C
ASD-21	UF	AS	738	24.5142003	3036	2985	0.0569462	0.4062	0.397949	P-C
ASD-21	UM	AS	909	27.49962184	3626	2985	0.258733	0.3836	0.384449	P-C
ASD-24	UF	UM	129	7.234997196	1689	1877	-0.0587144	0.3533	0.305921	NC
ASD-24	UF	AS	309	19.50142001	1689	1480	0.323772	0.3846	0.39661	P-C
ASD-24	UM	AS	361	21.50729818	1877	1480	0.17868	0.3846	0.370079	P-C
ASD-37	UF	UM	66	3.774663998	1483	2014	0.00739679	0.3388	0.329085	NC
ASD-37	UF	AS	283	18.42447917	1483	1589	0.294294	0.385	0.386174	P-C
ASD-37	UM	AS	376	20.87149598	2014	1589	0.267167	0.3768	0.395785	P-C
ASD-9	UF	AS	380	81.77833438	1582	1612	0.281	0.4954	0.483701	P-C
ASD-9	UM	AS	354	5.893657912	1590	1540	0.2793	0.4954	0.291398	P-C
ASD-9	UM	UF	89	5.647208122	1612	1540	-0.0534879	0.3363	0.287406	NC
ASD-38	UF	UM	158	8.873911823	1623	1938	-0.0247468	0.356	0.314678	C_L
ASD-38	UF	AS	396	23.94919867	1623	1684	0.27533	0.39	0.386339	P-C
ASD-38	UM	AS	392	21.64549972	1938	1684	0.298548	0.3851	0.399113	P-C
ASD-16	UF	UM	177	11.79213857	1461	1541	0.0292522	0.3603	0.307292	C
ASD-16	UF	AD	484	29.0080911	1461	1876	0.449547	0.3883	0.391919	P-C
ASD-16	UM	AD	454	26.57301727	1541	1876	0.438091	0.3824	0.379276	P-C
ASD-55	UF	UM	129	6.51350669	2110	1851	-0.0242128	0.3467	0.305648	NC
ASD-55	UF	AS	377	19.48823986	2110	1759	0.244642	0.3825	0.388761	P-C
ASD-55	UM	AS	396	21.93905817	1851	1759	0.286852	0.379	0.396141	P-C
ASD-73	UF	UM	162	8.061706892	2275	1744	-0.0146583	0.3446	0.318872	C_L
ASD-73	UF	AS	471	24.0490171	2275	1642	0.262493	0.3723	0.388697	P-C
ASD-73	UM	AS	369	21.79562906	1744	1642	0.260935	0.3881	0.387458	P-C

Homo member: total number of homozygous variants. %Shared Homozygosity= shared homozygosity/[(homo member1+homo member2)/2]X100.
Kinship (KING Program) and Kinship2 implemented by (VCFtools). AS: affected son. UM: unaffected mother. UF: unaffected father. P-C: parent-child. C_L: possible consanguinity. C: consanguinity. Relatedness is denoted as not confirmed (NC) if shared homo<148. Expected A_{jk} statistics of zero for individuals within a population and of one for MZ twins/duplicates.

Supplementary Table S4. Predictive mutation assessment software scores.

Proband ID/(Gender)	Gene	Base Change	Amino Acid Change	SIFT	PolyPhen 2	Mutation Taster
ASD-9 (M)	*AGL*	c.294-2A>T	NA	NA	NA	disease causing
	APC2	c.1207+3G>C	NA	NA	NA	disease causing
ASD-16 (F)	*MAN1B1*	c.1897G>T	p.V633F	0	1/D	disease causing
	KCTD21	c.152G>A	p.R51H	0.001	0.998/D	disease causing
	DUSP3	c.439G>A	p.V147I	0.05	0.979/D	disease causing
	CXorf30/CFAP47	c.8943G>A	p.M2981I	0.02	0.006/B	polymorphism
	SSX3	c.268C>A	p.R90S	0.04	0.097/B	polymorphism
	AVPR2	c.739-750 delCGCCGCAGGGGA	p.247-250del	NA	NA	polymorphism
ASD-17 (M)	*CRY1*	c.272G>A	p.W91X	NA	NA	disease causing
	NLRP2	c.523T>C	p.W175R	0.14	0.666/P	polymorphism
	ACE2	c.1235 C>A	p.A412E	0.006	0.991/D	disease causing
ASD-18 (M)	*GLT8D1*	c.811_812+2delGAGT	p.E271K	NA	NA	disease causing
	DNAJC13	c.5594A>G	p.N1865S	0.109	0.945/D	disease causing
	OR6C65	c.766delA	p.M256fs	NA	NA	disease causing
	HSPBP1	c.1016A>C	p.E339A	0.01	0.959/D	disease causing
	ATP2B3	c.1678C>G	p.P560A	0.878	0/B	polymorphism
ASD-19 (M)	*MXRA5*	c.28C>T	p.L10F	0.007	0.997/D	polymorphism
	ITIH6	c.1153A>G	p.S385G	0.527	0.001/B	polymorphism
	KDM5B	c.2265C>A	p.Y755X	NA	NA	disease causing
ASD-21 (M)	*NT5DC1*	c.1114T>C	p.S372P	0.007	0.998/D	disease causing
	TRIM9	c.1643G>A	p.R548H	0	0.856/P	disease causing
ASD-24 (M)	*GPKOW*	c.482C>T	p.A161V	0.294	0.008/B	polymorphism
	HTATSF1	c.894T>G	p.F298L	0.243	0.999/D	disease causing
	MOGS	c.832_833delAA	p.K278Efs*5	NA	NA	disease causing
ASD-37(M)	*IDS*	c.525T>A	p.D175E	0.17	0.94/P	disease causing
ASD-38 (M)	*ASB9*	c.310A>C	p.T104P	0.001	0.997/D	disease causing
	PDK3	c.248+3A>G	_	NA	NA	disease causing
	PLP1	c.328G>A	p.G110S	0.002	0.996/D	disease causing
ASD-39 (M)	*NEB*	c.5939T>C	p.L1980S	0.114	1/D	disease causing
ASD-40 (M)	*BOC*	c.1624G>A	p.G542R	0.009	0.987/D	disease causing
	SSTR3	c.724G>C	p.A242P	NA	0.987/D	disease causing
ASD-43 (M)	*NGF*	c.361C>T	p.R121W	0	1/D	disease causing
	SUMF1	c.710A>G	p.Q237R	0.03	1/D	disease causing
ASD-58 (M)	*MAOB*	c.392G>T	p.S131I	0.186	0.234/B	polymorphism
	ZNF630	c.77A>T	p.N26I	0	0.454/P	polymorphism
	PRODH2	c.625G>C	p.G209R	0.437	0/B	polymorphism
ASD-64 (F)	*USP9X*	c.3803A>G	p.Y1268C	0.036	0.007/B	disease causing

	RPS6KA6	c.1535A>G	p.Q512R	0.195	0.195/B	disease causing
	DDX26B/ INTS6L	c.1304A>T	p.E435V	0.022	0.843/P	disease causing
ASD-66 (M)	*SEMG2*	c.500G>A	p.W167X	NA	NA	disease causing
ASD-69 (M)	*CELSR2*	c.8185G>C	p.D2729H	0.115	0.999/D	disease causing
	1ITIH2	c.1863_1864insTATT	p.V622Yfs*17	NA	NA	disease causing
	CEP152	c.1718A>C	p.D573A	0.123	0.775/P	polymorphism
	ARSH	c.1124T>C	p.I375T	0.001	0.995/D	polymorphism
ASD-73 (M)	*FGF5*	c.251C>T	p.S84L	0.034	1/D	disease causing
	SMS	c.424G>T	p.L142F	0.04	0.559/P	disease causing
	FLNA	c.7079T>C	p.V2360A	0.024	0.971/D	disease causing

Polyphen2 scores: Benign (B), Probably damaging (D), possibly damaging (P). NA: not applicable.

Supplementary Table S5. Categories found overrepresented as identified by IPA.

Category	p-value range	#Molecules	Molecules
Top Diseases and disorders			
Hereditary Disorder	4.42E-02 - 1.37E-07	17	ATP2B3,AVPR2,FLNA,IDS,MAOB,PDK3,PLP1,SMS,USP9X,SUMF1,SSTR3, CEP152,MAN1B1,MOGS,NGF,NEB,AGL
Organismal Injury and Abnormalities	4.42E-02 - 1.37E-07	45	ACE2,AGL,APC2,ARSH,ASB9,ATP2B3,AVPR2,BOC,CELSR2,CEP152,CFAP47,CRY1, DNAJC13,DUSP3,FGF5,FLNA,GLT8D1,GPKOW,HSPBP1,HTATSF1, IDS,INTS6L, ITIH2,KCTD21,KDM5B,MAN1B1,MAOB, MOGS,MXRA5,NEB,NGF,NLRP2,NT5DC1,OR6C65,PDK3,PLP1,PRODH2, RPS6KA6,SEMG2, SMS,SSTR3,SSX3,SUMF1,TRIM9, USP9X
Cancer	3.98E-02- 4.63E-06	45	ACE2,AGL,APC2,ARSH,ASB9,ATP2B3,AVPR2,BOC,CELSR2,CEP152,CFAP47,CRY1, DNAJC13,DUSP3,FGF5,FLNA,GLT8D1,GPKOW,HSPBP1,HTATSF1, IDS,INTS6L, ITIH2,KCTD21,KDM5B,MAN1B1,MAOB, MOGS,MXRA5,NEB,NGF,NLRP2,NT5DC1,OR6C65,PDK3,PLP1,PRODH2, RPS6KA6,SEMG2, SMS,SSTR3,SSX3,SUMF1,TRIM9, USP9X
Gastrointestinal Disease	3.98E-02- 4.63E-06	44	ACE2,AGL,APC2,ASB9,ATP2B3,AVPR2,BOC,CELSR2,CEP152,CFAP47, CRY1,DNAJC13, DUSP3,FGF5,FLNA,GLT8D1,GPKOW,HSPBP1,HTATSF1,IDS,INTS6L,ITIH2, KCTD21,KDM5B,MAN1B1,MAOB,MOGS, MXRA5,NEB, NGF,NLRP2,NT5DC1,OR6C65,PDK3,PLP1,PRODH2,RPS6KA6,SEMG2,SMS,SSTR3,SSX3, SUMF1,TRIM9, USP9X
Neurological Disease	3.98E-02- 2.10E-05	18	AGL,ATP2B3,AVPR2,DNAJC13,IDS,MAOB,NEB,NGF,PLP1,MAN1B1, CEP152,PDK3,USP9X,FLNA,SMS,SEMG2, BOC,FGF5
Top molecular and Cellular Functions			
Cell Signaling	2.67E-02- 2.47E-04	8	AVPR2,CRY1,NGF,SSTR3,MAOB,PLP1,ACE2,FLNA
Nucleic Acid Metabolism	2.89E-02- 2.47E-04	5	AVPR2,CRY1,NGF,SSTR3,HSPBP1
Small Molecule Biochemistry	4.61E-02- 2.47E-04	12	AVPR2,CRY1,NGF,SSTR3,FGF5,MAOB,PLP1,ACE2,SMS,HSPBP1,KDM5B,ITIH2
Cell Morphology	4.42E-02- 1.02E-03	11	ATP2B3, BOC,CELSR2,NGF,NEB, SSTR3,TRIM9,FGF5,FLNA,PLP1,USP9X,
Cellular Assembly and Organization	4.42E-02- 1.02E-03	9	BOC,CELSR2,FLNA,NGF,SSTR3,TRIM9, NEB,USP9X, PLP1
Top Physiological System Development and Function			
Nervous System Development and Function	4.42E-02- 1.02E-03	11	BOC,CELSR2,FLNA,NGF,SSTR3,TRIM9,FGF5,PLP1,CRY1,MAOB,USP9X
Tissue Development	4.20E-02- 1.02E-03	11	BOC,CELSR2,FLNA,NGF,SSTR3,TRIM9,KDM5B,PLP1,FGF5,USP9X,AVPR2
Embryonic Development	4.20E-02- 2.26E-03	8	NGF,SSTR3,TRIM9,FLNA,USP9X,AVPR2,CELSR2,KDM5B
Hematological System Development and Function	4.20E-02- 2.26E-03	5	PLP1,AVPR2,NGF,FLNA,ACE2
Organ Development	4.20E-02- 2.26E-03	8	FGF5,NGF,NEB,AVPR2,KDM5B,FLNA,PLP1,USP9X

IPA (Version 01-07, https://analysis.ingenuity.com/pa/installer/select)

Supplementary Table S6. Clinical and demographic information for some of the patients.

Proband-ID	Gender	Consanguinity (Y/N)	ADOS (Y/N)	ADI-R (Y/N)	DSM-IV-TR	Other Intelligence and Cognitive measures (score)	Other developmental tests (score)	Language delay (Y/N)	Regression (Y/N)	Other clinical features	Other medical conditions
ASD-9	M	N	Y	Y	Y	NR	NR	NR	NR	NR	NR
ASD-16	F	Y*	Y	Y	Y	LIPS (52)	NR	Y	NR	severe social delay	NR
ASD-17	M	Y*	Y	Y	Y	WISC-IV (MA=52/CA=132)	NR	Y	NR	NR	G6PD def.
ASD-18	M	N	Y	Y	Y	LIPS (55)	NR	Y	NR	hyperactive/impulsive	NR
ASD-19	M	N	Y	Y	Y	NR	Bayleys (MA=20/CA=38)	Y	Y	NR	NR
ASD-21	M	Y*	Y	Y	Y	LIPS (100)	NR	Y	NR	hyperactive/impulsive	NR
ASD-24	M	N	Y	Y	Y	NR	Bayleys (MA=24 /CA=28)	N	NR	NR	NR
ASD-37	M	N	Y	Y	Y	NR	NR	Y	NR	NR	NR
ASD-38	M	Y*	Y	Y	Y	NR	NR	N	Y	NR	NR
ASD-39	M	Y*	Y	Y	Y	LIPS (68)	NR	Y	N	hyperactive/impulsive	NR
ASD-40	M	Y*	Y	Y	Y	NR	NR	Y	Y	hyperactive/impulsive	NR
ASD-43	M	N	Y	Y	Y	NR	NR	Y	NR	NR	NR
ASD-52	M	N	Y	Y	Y	NR	NR	Y	NR	hyperactive/impulsive	NR
ASD-55	M	N	Y	Y	Y	NR	NR	Y	N	microcephalic/cerebral atrophy	NR
ASD-58	M	N	Y	Y	Y	NR	NR	Y	NR	NR	NR
ASD-64	F	N	Y	Y	Y	NR	Bayleys (MA=13/CA=60)	Y	Y	NR	NR
ASD-66	M	Y*	Y	Y	Y	LIPS (62)	NR	Y	Y	hyperactive/impulsive	NR
ASD-69	M	Y*	Y	Y	Y	NR	NR	NR	NR	NR	con. Heart dis.
ASD-73	M	Y*	Y	Y	Y	NR	NR	NR	NR	NR	NR

LIPS: Leiter International Performance Scale. **WISC-IV:** Wechsler Intelligence Scale for Children version 4. **Bayleys :** Bayley Scales of Infant Development. **MA :** Mental Age in months. CA: Chronological age in months. **G6PDH def:** Glucose-6-phosphate dehydrogenase deficiency. **con. Heart dis:** Congenital Heart Disease. *validated by relatedness analysis.

Supplementary Table S7. List of genes/loci investigated for CNVs.

Physical location	Candidate Genes	OMIM#
chr2:49,916,505-51,034,536	*NRXN1*	600565
chr2:114,440,322-115,846,750	*DPP10*	608209
chr2:211,373,717-212,540,628	*ERBB4*	600543
chr3:2,096,866-3,059,961	*CNTN4*	607280
chr3:6,859,115-7,743,531	*GRM7*	604101
chr3:173,396,448-174,285,349	*NLGN1*	600568
chr4:46,033,770-46,126,065	*GABRG1, GABRA4, GABRA2*	137166, 137141, 137140
chr5:36,604,355-36,690,334	*SLC1A3*	600111
chr7:116,951,327-117,232,021	*ST7*	600833
chr7:146,114,361-148,422,996	*CNTNAP2*	604569
chr8:31,637,752-32,745,252	*NRG1*	142445
chr8:1,499,366-1,710,476	*DLGAP2*	605438
chr8:140,656,382-141,003,313	*PTK2*	600758
chr11:83,453,013-85,629,270	*DLG2*	603583
chr12:66,345,431-66,681,145	*GRIP1*	604597
chr15:26,864,719-26,951,210	*GABRA5, GABRB3, GABRG3*	137142, 137192, 600233
chr15:28,919,637-29,120,313	*APBA2*	602712
chr15:32,028,483-32,172,183	*CHRNA7*	118511
chr16:30,003,514-30,015,596	*DOC2A*	604567
chr17:45,892,382-46,030,333	*MAPT*	157140
chr22:18,910,774-18,938,553	*PRODH*	606810
chr22:50,672,642-50,735,212	*SHANK3*	606230
chrX:5,888,026-6,230,882	*NLGN4*	300427
chrX:38,559,478-38,690,918	*TSPAN7*	300096
chrX:41,512,936-41,925,034	*CASK*	300172
chrX:28,585,564-29,957,900	*IL1RAPL1*	300206
chrX:123,182,243-123,492,915	*GRIA3*	305915
chrX:154,019,813-154,099,731	*MECP2*	300005

Supplementary Figures

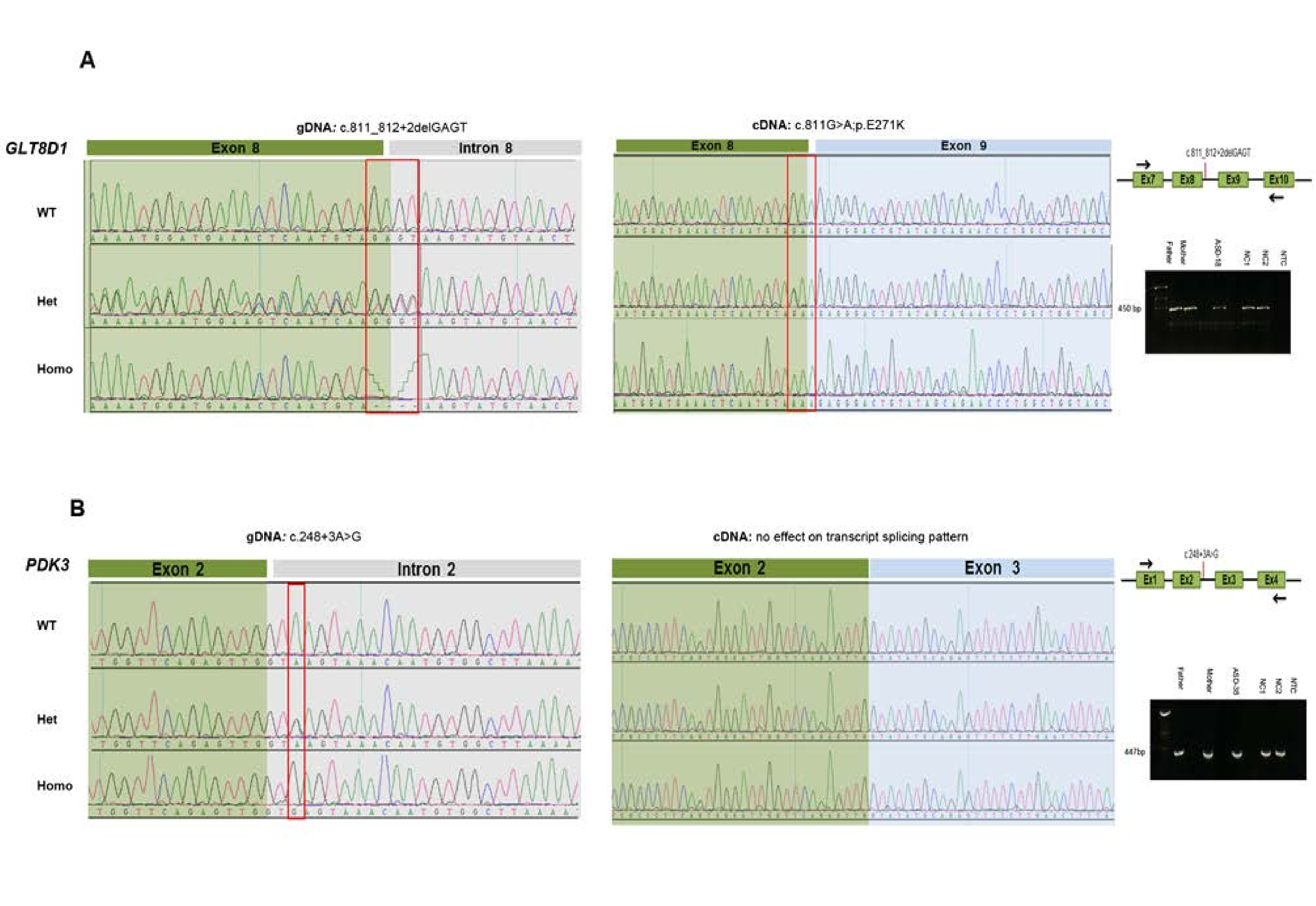

Supplementary Figure S1. Reverse transcription (RT)-PCR and Sanger analysis of homozygous splice site variants **of *GLT8D1* and *PDK3*.** (A) The effect of c.811_812+2delGAGT on *GLT8D1* splicing. Left, partial chromatogram displaying a deletion of 4 nucleotides spanning coding and intronic sequence in exon 8/intron 8 of *GLT8D1* detected in ASD-18 genomic DNA (gDNA). Middle, partial sequencing of *GLT8D1* transcript exons 8 and 9 showing the amino acid substitution (p.E271K) introduced by the variant. The 450bp long transcript was generated using the primer pair illustrated (right) and visualized on 2% Agarose gel. (B) The effect of c.248+3A>G splice donor variant on *PDK3* splicing. Left, partial chromatogram of the splice-site variant identified in ASD-38 gDNA. Middle, partial sequencing of *PDK3* transcript exons 2 and 3 indicating normal splicing pattern of ASD-38 mRNA, however, the possibility of the variant being leaky cannot be ruled out as normal transcript abundance has not been examined here. The 447bp long transcript was generated using the primer pair illustrated (right) and visualized on 2% Agarose gel. cDNA: coding DNA. Arrows point to forward primer and reverse primer locations. NC: normal control. NTC: no DNA template control.

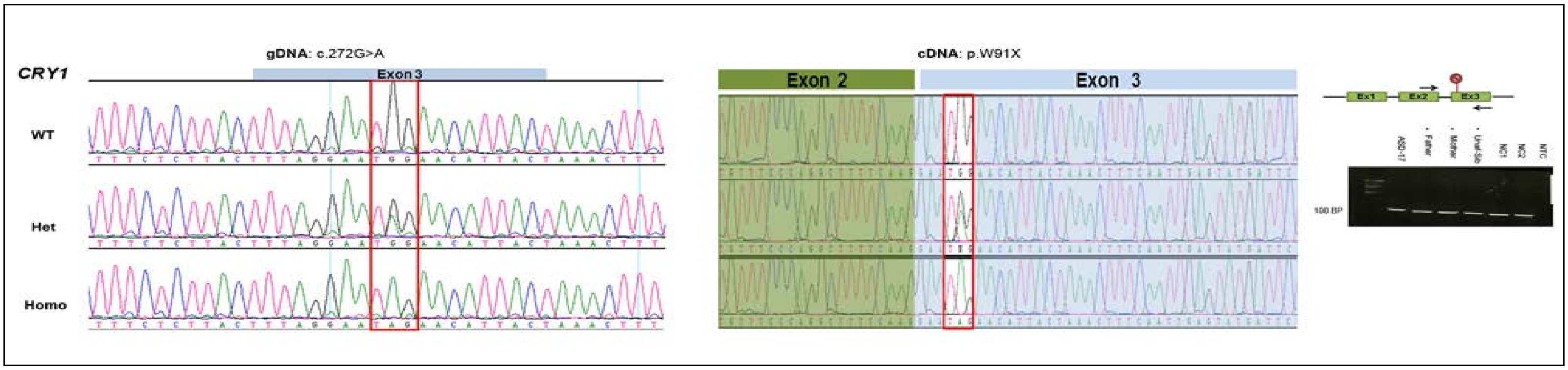

Supplementary Figure S2. Reverse transcription (RT)-PCR and Sanger analysis of homozygous nonsense variant **of CRY1.** c.272G>A nonsense variant of CRY1identifed in ASD-17. Sanger sequencing reveals the variant in genomic (left) and coding DNA (middle). The 100 bp transcript was generated using the primer pair illustrated (right) and was present in ASD-17 as shown by the gel electrophoresis image. The presence of a stable mRNA in the proband indicates that the variant does not activate nonsense-mediated mRNA decay and therefore may result in the formation of abnormal protein. gDNA: genomic DNA. cDNA: coding DNA. Arrows point to forward primer and reverse primer locations. Unaf-Sib: unaffected sibling. NC: normal control. NTC: no DNA template control. (*) denotes heterozygous for the change.

References

1 Shintani, T. *et al.* APC2 plays an essential role in axonal projections through the regulation of microtubule stability. *The Journal of neuroscience : the official journal of the Society for Neuroscience* **29**, 11628-11640, doi:10.1523/jneurosci.2394-09.2009 (2009).

2 Almuriekhi, M. *et al.* Loss-of-Function Mutation in APC2 Causes Sotos Syndrome Features. *Cell reports*, doi:10.1016/j.celrep.2015.02.011 (2015).

3 Najmabadi, H. *et al.* Deep sequencing reveals 50 novel genes for recessive cognitive disorders. *Nature* **478**, 57-63, doi:10.1038/nature10423 (2011).

4 Rafiq, M. A. *et al.* Mutations in the alpha 1,2-mannosidase gene, MAN1B1, cause autosomal-recessive intellectual disability. *American journal of human genetics* **89**, 176-182, doi:10.1016/j.ajhg.2011.06.006 (2011).

5 Rymen, D. *et al.* MAN1B1 deficiency: an unexpected CDG-II. *PLoS genetics* **9**, e1003989, doi:10.1371/journal.pgen.1003989 (2013).

6 Van Scherpenzeel, M. *et al.* Diagnostic serum glycosylation profile in patients with intellectual disability as a result of MAN1B1 deficiency. *Brain : a journal of neurology* **137**, 1030-1038, doi:10.1093/brain/awu019 (2014).

7 Iannotti, M. J., Figard, L., Sokac, A. M. & Sifers, R. N. A Golgi-localized mannosidase (MAN1B1) plays a non-enzymatic gatekeeper role in protein biosynthetic quality control. *The Journal of biological chemistry* **289**, 11844-11858, doi:10.1074/jbc.M114.552091 (2014).

8 Liu, Z., Xiang, Y. & Sun, G. The KCTD family of proteins: structure, function, disease relevance. *Cell & bioscience* **3**, 45, doi:10.1186/2045-3701-3-45 (2013).

9 De Smaele, E. *et al.* Identification and characterization of KCASH2 and KCASH3, 2 novel Cullin3 adaptors suppressing histone deacetylase and Hedgehog activity in medulloblastoma. *Neoplasia (New York, N.Y.)* **13**, 374-385 (2011).

10 Need, A. C. *et al.* A genome-wide investigation of SNPs and CNVs in schizophrenia. *PLoS genetics* **5**, e1000373, doi:10.1371/journal.pgen.1000373 (2009).

11 Pavic, K., Duan, G. & Kohn, M. VHR/DUSP3 phosphatase: structure, function and regulation. *The FEBS journal* **282**, 1871-1890, doi:10.1111/febs.13263 (2015).

12 Amand, M. *et al.* DUSP3/VHR is a pro-angiogenic atypical dual-specificity phosphatase. *Molecular cancer* **13**, 108, doi:10.1186/1476-4598-13-108 (2014).

13 Subramaniam, S. & Unsicker, K. ERK and cell death: ERK1/2 in neuronal death. *The FEBS journal* **277**, 22-29, doi:10.1111/j.1742-4658.2009.07367.x (2010).

14 Yamasaki, T., Kawasaki, H. & Nishina, H. Diverse Roles of JNK and MKK Pathways in the Brain. *Journal of signal transduction* **2012**, 459265, doi:10.1155/2012/459265 (2012).

15 Sanders, S. J. *et al.* De novo mutations revealed by whole-exome sequencing are strongly associated with autism. *Nature* **485**, 237-241, doi:10.1038/nature10945 (2012).

16 Zendman, A. J., Ruiter, D. J. & Van Muijen, G. N. Cancer/testis-associated genes: identification, expression profile, and putative function. *Journal of cellular physiology* **194**, 272-288, doi:10.1002/jcp.10215 (2003).

17 Huang, L., Poke, G., Gecz, J. & Gibson, K. A novel contiguous gene deletion of AVPR2 and ARHGAP4 genes in male dizygotic twins with nephrogenic diabetes insipidus and intellectual disability. *American journal of medical genetics. Part A* **158A**, 2511-2518, doi:10.1002/ajmg.a.35591 (2012).

18 Meyer-Lindenberg, A., Domes, G., Kirsch, P. & Heinrichs, M. Oxytocin and vasopressin in the human brain: social neuropeptides for translational medicine. *Nature reviews. Neuroscience* **12**, 524-538, doi:10.1038/nrn3044 (2011).

19 Spanakis, E., Milord, E. & Gragnoli, C. AVPR2 variants and mutations in nephrogenic diabetes insipidus: review and missense mutation significance. *Journal of cellular physiology* **217**, 605-617, doi:10.1002/jcp.21552 (2008).

20 Cortesi, F., Giannotti, F., Ivanenko, A. & Johnson, K. Sleep in children with autistic spectrum disorder. *Sleep medicine* **11**, 659-664, doi:10.1016/j.sleep.2010.01.010 (2010).

21 Rosenwasser, A. M. Circadian clock genes: non-circadian roles in sleep, addiction, and psychiatric disorders? *Neuroscience and biobehavioral reviews* **34**, 1249-1255, doi:10.1016/j.neubiorev.2010.03.004 (2010).
22 Fontalba, A., Gutierrez, O. & Fernandez-Luna, J. L. NLRP2, an inhibitor of the NF-kappaB pathway, is transcriptionally activated by NF-kappaB and exhibits a nonfunctional allelic variant. *Journal of immunology (Baltimore, Md. : 1950)* **179**, 8519-8524 (2007).
23 Minkiewicz, J., de Rivero Vaccari, J. P. & Keane, R. W. Human astrocytes express a novel NLRP2 inflammasome. *Glia* **61**, 1113-1121, doi:10.1002/glia.22499 (2013).
24 Meyer, E. *et al.* Germline mutation in NLRP2 (NALP2) in a familial imprinting disorder (Beckwith-Wiedemann Syndrome). *PLoS genetics* **5**, e1000423, doi:10.1371/journal.pgen.1000423 (2009).
25 Guimond, M. O. & Gallo-Payet, N. The Angiotensin II Type 2 Receptor in Brain Functions: An Update. *International journal of hypertension* **2012**, 351758, doi:10.1155/2012/351758 (2012).
26 Burrell, L. M., Harrap, S. B., Velkoska, E. & Patel, S. K. The ACE2 gene: its potential as a functional candidate for cardiovascular disease. *Clinical science (London, England : 1979)* **124**, 65-76, doi:10.1042/cs20120269 (2013).
27 Sasayama, D. *et al.* ITIH3 polymorphism may confer susceptibility to psychiatric disorders by altering the expression levels of GLT8D1. *Journal of psychiatric research* **50**, 79-83, doi:10.1016/j.jpsychires.2013.12.002 (2014).
28 Gustavsson, E. K. *et al.* DNAJC13 genetic variants in parkinsonism. *Movement disorders : official journal of the Movement Disorder Society* **30**, 273-278, doi:10.1002/mds.26064 (2015).
29 Patak, J., Zhang-James, Y. & Faraone, S. V. Endosomal system genetics and autism spectrum disorders: A literature review. *Neuroscience and biobehavioral reviews* **65**, 95-112, doi:10.1016/j.neubiorev.2016.03.022 (2016).
30 Freeman, C. L., Hesketh, G. & Seaman, M. N. RME-8 coordinates the activity of the WASH complex with the function of the retromer SNX dimer to control endosomal tubulation. *Journal of cell science* **127**, 2053-2070, doi:10.1242/jcs.144659 (2014).
31 Murphy, M. E. The HSP70 family and cancer. *Carcinogenesis* **34**, 1181-1188, doi:10.1093/carcin/bgt111 (2013).
32 Vos, M. J., Hageman, J., Carra, S. & Kampinga, H. H. Structural and functional diversities between members of the human HSPB, HSPH, HSPA, and DNAJ chaperone families. *Biochemistry* **47**, 7001-7011, doi:10.1021/bi800639z (2008).
33 Morimoto, R. I. Proteotoxic stress and inducible chaperone networks in neurodegenerative disease and aging. *Genes & development* **22**, 1427-1438, doi:10.1101/gad.1657108 (2008).
34 Nishimura, Y. *et al.* Genome-wide expression profiling of lymphoblastoid cell lines distinguishes different forms of autism and reveals shared pathways. *Human molecular genetics* **16**, 1682-1698, doi:10.1093/hmg/ddm116 (2007).
35 Olender, T., Lancet, D. & Nebert, D. W. Update on the olfactory receptor (OR) gene superfamily. *Human genomics* **3**, 87-97 (2008).
36 Berko, E. R. *et al.* Mosaic epigenetic dysregulation of ectodermal cells in autism spectrum disorder. *PLoS genetics* **10**, e1004402, doi:10.1371/journal.pgen.1004402 (2014).
37 Ashwin, C. *et al.* Enhanced olfactory sensitivity in autism spectrum conditions. *Molecular autism* **5**, 53, doi:10.1186/2040-2392-5-53 (2014).
38 Rozenkrantz, L. *et al.* A Mechanistic Link between Olfaction and Autism Spectrum Disorder. *Current biology : CB* **25**, 1904-1910, doi:10.1016/j.cub.2015.05.048 (2015).
39 Brini, M. & Carafoli, E. The plasma membrane Ca(2)+ ATPase and the plasma membrane sodium calcium exchanger cooperate in the regulation of cell calcium. *Cold Spring Harbor perspectives in biology* **3**, doi:10.1101/cshperspect.a004168 (2011).
40 Pereira, R. R. *et al.* Screening for intellectual disability using high-resolution CMA technology in a retrospective cohort from Central Brazil. *PloS one* **9**, e103117, doi:10.1371/journal.pone.0103117 (2014).
41 Turkish, A. R. *et al.* Identification of two novel human acyl-CoA wax alcohol acyltransferases: members of the diacylglycerol acyltransferase 2 (DGAT2) gene superfamily. *The Journal of biological chemistry* **280**, 14755-14764, doi:10.1074/jbc.M500025200 (2005).
42 Nava, C. *et al.* Analysis of the chromosome X exome in patients with autism spectrum disorders identified novel candidate genes, including TMLHE. *Translational psychiatry* **2**, e179, doi:10.1038/tp.2012.102 (2012).
43 Barros, C. S., Franco, S. J. & Muller, U. Extracellular matrix: functions in the nervous system. *Cold Spring Harbor perspectives in biology* **3**, a005108, doi:10.1101/cshperspect.a005108 (2011).

44 Dey, B. K. *et al.* The histone demethylase KDM5b/JARID1b plays a role in cell fate decisions by blocking terminal differentiation. *Molecular and cellular biology* **28**, 5312-5327, doi:10.1128/mcb.00128-08 (2008).
45 Li, X. *et al.* Histone demethylase KDM5B is a key regulator of genome stability. *Proceedings of the National Academy of Sciences of the United States of America* **111**, 7096-7101, doi:10.1073/pnas.1324036111 (2014).
46 Ounap, K. *et al.* A novel c.2T > C mutation of the KDM5C/JARID1C gene in one large family with X-linked intellectual disability. *European journal of medical genetics* **55**, 178-184, doi:10.1016/j.ejmg.2012.01.004 (2012).
47 Athanasakis, E. *et al.* Next generation sequencing in nonsyndromic intellectual disability: from a negative molecular karyotype to a possible causative mutation detection. *American journal of medical genetics. Part A* **164A**, 170-176, doi:10.1002/ajmg.a.36274 (2014).
48 Iossifov, I. *et al.* The contribution of de novo coding mutations to autism spectrum disorder. *Nature* **515**, 216-221, doi:10.1038/nature13908 (2014).
49 Bigdeli, T. B. *et al.* Association study of 83 candidate genes for bipolar disorder in chromosome 6q selected using an evidence-based prioritization algorithm. *American journal of medical genetics. Part B, Neuropsychiatric genetics : the official publication of the International Society of Psychiatric Genetics* **162B**, 898-906, doi:10.1002/ajmg.b.32200 (2013).
50 Nurnberger, J. I., Jr. *et al.* Identification of pathways for bipolar disorder: a meta-analysis. *JAMA psychiatry* **71**, 657-664, doi:10.1001/jamapsychiatry.2014.176 (2014).
51 Butler, M. G., Rafi, S. K., Hossain, W., Stephan, D. A. & Manzardo, A. M. Whole exome sequencing in females with autism implicates novel and candidate genes. *International journal of molecular sciences* **16**, 1312-1335, doi:10.3390/ijms16011312 (2015).
52 Winkle, C. C. *et al.* A novel Netrin-1-sensitive mechanism promotes local SNARE-mediated exocytosis during axon branching. *The Journal of cell biology* **205**, 217-232, doi:10.1083/jcb.201311003 (2014).
53 Engle, E. C. Human genetic disorders of axon guidance. *Cold Spring Harbor perspectives in biology* **2**, a001784, doi:10.1101/cshperspect.a001784 (2010).
54 Zikopoulos, B. & Barbas, H. Changes in prefrontal axons may disrupt the network in autism. *The Journal of neuroscience : the official journal of the Society for Neuroscience* **30**, 14595-14609, doi:10.1523/jneurosci.2257-10.2010 (2010).
55 Zikopoulos, B. & Barbas, H. Altered neural connectivity in excitatory and inhibitory cortical circuits in autism. *Frontiers in human neuroscience* **7**, 609, doi:10.3389/fnhum.2013.00609 (2013).
56 Tanji, K. *et al.* TRIM9, a novel brain-specific E3 ubiquitin ligase, is repressed in the brain of Parkinson's disease and dementia with Lewy bodies. *Neurobiology of disease* **38**, 210-218, doi:10.1016/j.nbd.2010.01.007 (2010).
57 Miller, H. B., Robinson, T. J., Gordan, R., Hartemink, A. J. & Garcia-Blanco, M. A. Identification of Tat-SF1 cellular targets by exon array analysis reveals dual roles in transcription and splicing. *RNA (New York, N.Y.)* **17**, 665-674, doi:10.1261/rna.2462011 (2011).
58 Aksaas, A. K. *et al.* G-patch domain and KOW motifs-containing protein, GPKOW; a nuclear RNA-binding protein regulated by protein kinase A. *Journal of molecular signaling* **6**, 10, doi:10.1186/1750-2187-6-10 (2011).
59 Lyons, J. J., Milner, J. D. & Rosenzweig, S. D. Glycans Instructing Immunity: The Emerging Role of Altered Glycosylation in Clinical Immunology. *Frontiers in pediatrics* **3**, 54, doi:10.3389/fped.2015.00054 (2015).
60 Burrow, T. A. & Leslie, N. D. Review of the use of idursulfase in the treatment of mucopolysaccharidosis II. *Biologics : targets & therapy* **2**, 311-320 (2008).
61 Kennerson, M. L. *et al.* A new locus for X-linked dominant Charcot-Marie-Tooth disease (CMTX6) is caused by mutations in the pyruvate dehydrogenase kinase isoenzyme 3 (PDK3) gene. *Human molecular genetics* **22**, 1404-1416, doi:10.1093/hmg/dds557 (2013).
62 Wyss, M. & Kaddurah-Daouk, R. Creatine and creatinine metabolism. *Physiological reviews* **80**, 1107-1213 (2000).
63 Jost, C. R. *et al.* Creatine kinase B-driven energy transfer in the brain is important for habituation and spatial learning behaviour, mossy fibre field size and determination of seizure susceptibility. *The European journal of neuroscience* **15**, 1692-1706 (2002).
64 Streijger, F. *et al.* Structural and behavioural consequences of double deficiency for creatine kinases BCK and UbCKmit. *Behavioural brain research* **157**, 219-234, doi:10.1016/j.bbr.2004.07.002 (2005).
65 Nave, K. A. Myelination and the trophic support of long axons. *Nature reviews. Neuroscience* **11**, 275-283, doi:10.1038/nrn2797 (2010).

66 Fields, R. D. White matter in learning, cognition and psychiatric disorders. *Trends in neurosciences* **31**, 361-370, doi:10.1016/j.tins.2008.04.001 (2008).
67 Laitila, J. *et al.* Expression of multiple nebulin isoforms in human skeletal muscle and brain. *Muscle & nerve* **46**, 730-737, doi:10.1002/mus.23380 (2012).
68 Lehtokari, V. L. *et al.* Identification of 45 novel mutations in the nebulin gene associated with autosomal recessive nemaline myopathy. *Human mutation* **27**, 946-956, doi:10.1002/humu.20370 (2006).
69 Scoto, M. *et al.* Nebulin (NEB) mutations in a childhood onset distal myopathy with rods and cores uncovered by next generation sequencing. *European journal of human genetics : EJHG* **21**, 1249-1252, doi:10.1038/ejhg.2013.31 (2013).
70 Jin, H. S. *et al.* Identification of the rare compound heterozygous variants in the NEB gene in a Korean family with intellectual disability, epilepsy and early-childhood-onset generalized muscle weakness. *Journal of human genetics* **59**, 643-647, doi:10.1038/jhg.2014.87 (2014).
71 Courchet, J. & Polleux, F. Sonic hedgehog, BOC, and synaptic development: new players for an old game. *Neuron* **73**, 1055-1058, doi:10.1016/j.neuron.2012.03.008 (2012).
72 Quintela, I. *et al.* Female patient with autistic disorder, intellectual disability, and co-morbid anxiety disorder: Expanding the phenotype associated with the recurrent 3q13.2-q13.31 microdeletion. *American journal of medical genetics. Part A* **167**, 3121-3129, doi:10.1002/ajmg.a.37292 (2015).
73 Guadiana, S. M. *et al.* Arborization of dendrites by developing neocortical neurons is dependent on primary cilia and type 3 adenylyl cyclase. *The Journal of neuroscience : the official journal of the Society for Neuroscience* **33**, 2626-2638, doi:10.1523/jneurosci.2906-12.2013 (2013).
74 Einstein, E. B. *et al.* Somatostatin signaling in neuronal cilia is critical for object recognition memory. *The Journal of neuroscience : the official journal of the Society for Neuroscience* **30**, 4306-4314, doi:10.1523/jneurosci.5295-09.2010 (2010).
75 Gibbs, T. T., Russek, S. J. & Farb, D. H. Sulfated steroids as endogenous neuromodulators. *Pharmacology, biochemistry, and behavior* **84**, 555-567, doi:10.1016/j.pbb.2006.07.031 (2006).
76 Baranzini, S. E. *et al.* Genetic variation influences glutamate concentrations in brains of patients with multiple sclerosis. *Brain : a journal of neurology* **133**, 2603-2611, doi:10.1093/brain/awq192 (2010).
77 Gai, X. *et al.* Rare structural variation of synapse and neurotransmission genes in autism. *Molecular psychiatry* **17**, 402-411, doi:10.1038/mp.2011.10 (2012).
78 Glessner, J. T. *et al.* Autism genome-wide copy number variation reveals ubiquitin and neuronal genes. *Nature* **459**, 569-573, doi:10.1038/nature07953 (2009).
79 Capsoni, S. From genes to pain: nerve growth factor and hereditary sensory and autonomic neuropathy type V. *The European journal of neuroscience* **39**, 392-400, doi:10.1111/ejn.12461 (2014).
80 Berry, A., Bindocci, E. & Alleva, E. NGF, brain and behavioral plasticity. *Neural plasticity* **2012**, 784040, doi:10.1155/2012/784040 (2012).
81 Kana, R. K., Uddin, L. Q., Kenet, T., Chugani, D. & Muller, R. A. Brain connectivity in autism. *Frontiers in human neuroscience* **8**, 349, doi:10.3389/fnhum.2014.00349 (2014).
82 Camarena, B. *et al.* Monoamine oxidase a and B gene polymorphisms and negative and positive symptoms in schizophrenia. *ISRN psychiatry* **2012**, 852949, doi:10.5402/2012/852949 (2012).
83 Wei, Y. L., Li, C. X., Li, S. B., Liu, Y. & Hu, L. Association study of monoamine oxidase A/B genes and schizophrenia in Han Chinese. *Behavioral and brain functions : BBF* **7**, 42, doi:10.1186/1744-9081-7-42 (2011).
84 Gasso, P. *et al.* Association of A/G polymorphism in intron 13 of the monoamine oxidase B gene with schizophrenia in a Spanish population. *Neuropsychobiology* **58**, 65-70, doi:10.1159/000159774 (2008).
85 Salem, A. M. *et al.* Genetic variants of neurotransmitter-related genes and miRNAs in Egyptian autistic patients. *TheScientificWorldJournal* **2013**, 670621, doi:10.1155/2013/670621 (2013).
86 Bortolato, M. *et al.* Monoamine oxidase A and A/B knockout mice display autistic-like features. *The international journal of neuropsychopharmacology / official scientific journal of the Collegium Internationale Neuropsychopharmacologicum (CINP)* **16**, 869-888, doi:10.1017/s1461145712000715 (2013).
87 Maynard, T. M. *et al.* A comprehensive analysis of 22q11 gene expression in the developing and adult brain. *Proceedings of the National Academy of Sciences of the United States of America* **100**, 14433-14438, doi:10.1073/pnas.2235651100 (2003).

88 Liu, H. *et al.* Genetic variation at the 22q11 PRODH2/DGCR6 locus presents an unusual pattern and increases susceptibility to schizophrenia. *Proceedings of the National Academy of Sciences of the United States of America* **99**, 3717-3722, doi:10.1073/pnas.042700699 (2002).

89 Homan, C. C. *et al.* Mutations in USP9X are associated with X-linked intellectual disability and disrupt neuronal cell migration and growth. *American journal of human genetics* **94**, 470-478, doi:10.1016/j.ajhg.2014.02.004 (2014).

90 Jolly, L. A., Taylor, V. & Wood, S. A. USP9X enhances the polarity and self-renewal of embryonic stem cell-derived neural progenitors. *Molecular biology of the cell* **20**, 2015-2029, doi:10.1091/mbc.E08-06-0596 (2009).

91 Cargnello, M. & Roux, P. P. Activation and function of the MAPKs and their substrates, the MAPK-activated protein kinases. *Microbiology and molecular biology reviews : MMBR* **75**, 50-83, doi:10.1128/mmbr.00031-10 (2011).

92 Lundwall, A., Bjartell, A., Olsson, A. Y. & Malm, J. Semenogelin I and II, the predominant human seminal plasma proteins, are also expressed in non-genital tissues. *Molecular human reproduction* **8**, 805-810 (2002).

93 Berti, A. *et al.* Expression of seminal vesicle-specific antigen in serum of lung tumor patients. *Journal of forensic sciences* **50**, 1114-1115 (2005).

94 Rodrigues, R. G. *et al.* Semenogelins are ectopically expressed in small cell lung carcinoma. *Clinical cancer research : an official journal of the American Association for Cancer Research* **7**, 854-860 (2001).

95 Qu, Y. *et al.* Genetic evidence that Celsr3 and Celsr2, together with Fzd3, regulate forebrain wiring in a Vangl-independent manner. *Proceedings of the National Academy of Sciences of the United States of America* **111**, E2996-3004, doi:10.1073/pnas.1402105111 (2014).

96 Shima, Y., Kengaku, M., Hirano, T., Takeichi, M. & Uemura, T. Regulation of dendritic maintenance and growth by a mammalian 7-pass transmembrane cadherin. *Developmental cell* **7**, 205-216, doi:10.1016/j.devcel.2004.07.007 (2004).

97 Kalay, E. *et al.* CEP152 is a genome maintenance protein disrupted in Seckel syndrome. *Nature genetics* **43**, 23-26, doi:10.1038/ng.725 (2011).

98 Lindholm, D. *et al.* Fibroblast growth factor-5 promotes differentiation of cultured rat septal cholinergic and raphe serotonergic neurons: comparison with the effects of neurotrophins. *The European journal of neuroscience* **6**, 244-252 (1994).

99 Reuss, B., Dono, R. & Unsicker, K. Functions of fibroblast growth factor (FGF)-2 and FGF-5 in astroglial differentiation and blood-brain barrier permeability: evidence from mouse mutants. *The Journal of neuroscience : the official journal of the Society for Neuroscience* **23**, 6404-6412 (2003).

100 Zhang, L. *et al.* MEK-ERK1/2-dependent FLNA overexpression promotes abnormal dendritic patterning in tuberous sclerosis independent of mTOR. *Neuron* **84**, 78-91, doi:10.1016/j.neuron.2014.09.009 (2014).

101 Turecek, R. *et al.* Intracellular spermine decreases open probability of N-methyl-D-aspartate receptor channels. *Neuroscience* **125**, 879-887, doi:10.1016/j.neuroscience.2004.03.003 (2004).

102 Cason, A. L. *et al.* X-linked spermine synthase gene (SMS) defect: the first polyamine deficiency syndrome. *European journal of human genetics : EJHG* **11**, 937-944, doi:10.1038/sj.ejhg.5201072 (2003).
